# Mechanisms of Viscous Media Effects on Elementary Steps of Bacterial Bioluminescent Reaction

**DOI:** 10.3390/ijms22168827

**Published:** 2021-08-17

**Authors:** Albert E. Lisitsa, Lev A. Sukovatyi, Sergey I. Bartsev, Anna A. Deeva, Valentina A. Kratasyuk, Elena V. Nemtseva

**Affiliations:** 1 Biophysics Department, Siberian Federal University, Svobodny 79, 660041 Krasnoyarsk, Russia; lsukovatyy@sfu-kras.ru (L.A.S.); bartsev@yandex.ru (S.I.B.); adeeva@sfu-kras.ru (A.A.D.); VKratasyuk@sfu-kras.ru (V.A.K.); enemtseva@sfu-kras.ru (E.V.N.); 2 The Institute of Biophysics SB RAS, Akademgorodok 50/50, 660036 Krasnoyarsk, Russia

**Keywords:** bacterial luciferase, non-steady-state reaction kinetics, viscosity, diffusion limitation

## Abstract

Enzymes activity in a cell is determined by many factors, among which viscosity of the microenvironment plays a significant role. Various cosolvents can imitate intracellular conditions in vitro, allowing to reduce a combination of different regulatory effects. The aim of the study was to analyze the media viscosity effects on the rate constants of the separate stages of the bacterial bioluminescent reaction. Non-steady-state reaction kinetics in glycerol and sucrose solutions was measured by stopped-flow technique and analyzed with a mathematical model developed in accordance with the sequence of reaction stages. Molecular dynamics methods were applied to reveal the effects of cosolvents on luciferase structure. We observed both in glycerol and in sucrose media that the stages of luciferase binding with flavin and aldehyde, in contrast to oxygen, are diffusion-limited. Moreover, unlike glycerol, sucrose solutions enhanced the rate of an electronically excited intermediate formation. The MD simulations showed that, in comparison with sucrose, glycerol molecules could penetrate the active-site gorge, but sucrose solutions caused a conformational change of functionally important αGlu175 of luciferase. Therefore, both cosolvents induce diffusion limitation of substrates binding. However, in sucrose media, increasing enzyme catalytic constant neutralizes viscosity effects. The activating effect of sucrose can be attributed to its exclusion from the catalytic gorge of luciferase and promotion of the formation of the active site structure favorable for the catalysis.

## 1. Introduction

Inside the cells, biochemical processes occur in the microenvironment that is heterogeneous both in composition and spatial organization, significantly affecting the rate and equilibria of enzymatic reactions [1]. To reveal the mechanisms underlying these effects, biochemical kinetics and equilibria are studied in well-characterized media, which are designed to imitate one or more components of the complexity of living cells. This approach aims to fill the gap between traditional in vitro studies and the attempts to analyze reactions in intact cells [2].

One of the components of intracellular complexity along with the presence of high local concentrations of various macromolecules is a microscopic viscosity [3]. Numerous studies have demonstrated that microviscosity plays an essential role in cellular biophysics controlling the rates of diffusion and bimolecular reactions within the cell interior [4,5]. In experiments in vitro, increased viscosity is simulated by the addition of cosolvents, usually small carbohydrates or polyols [6], which can affect the enzymatic process by various mechanisms, including: (i) a change in the structural and dynamic characteristics of proteins through preferential hydration/binding, mobility of protein domains, etc. [7,8]; (ii) diffusion control of the enzymatic reaction stages [9,10]; (iii) specific interactions such as competitive inhibition [11,12].

The role of diffusion for prokaryote metabolism has been intensively discussed [2]. There are several recorded cases when the diffusion coefficients have been of tremendous importance for the regulation of intracellular processes, either determining the rate of diffusion-limited reactions or being a decisive factor for the existence of the phenomena [13,14]. Thus, the study of diffusion-controlled biochemical processes is pertinent as a step towards understanding the principles of biomolecular systems functioning under in vivo conditions.

The extent to which the diffusion controls the reaction can be revealed from the dependence of the elementary kinetic constant on the solvent viscosity. Usually the rate–viscosity relationship is given by $k∼\eta^{-\delta }$, where the exponent δ varies from 0 to 1, and the case δ=1 corresponds to the diffusion limitation. However, there are examples of enhanced effect with δ>1 in various reactions examined in wide viscosity range [15]. This approach was used for studying numerous processes, including enzyme kinetics, protein folding, inter- and intramolecular electron transfer, etc. [15]. In the current work, we used it to study the diffusion control of bacterial bioluminescence, which has not been performed before.

**Figure 1:**
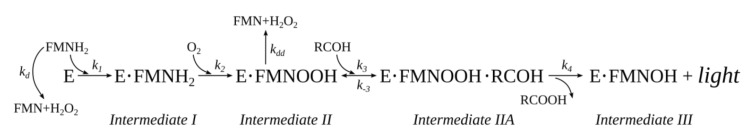
The stages of the reaction, catalyzed by bacterial luciferase. E—enzyme, FMN—flavin mononucleotide, O${}_{2}$—molecular oxygen, RCOH and RCOOH—long-chain aldehyde and corresponding carbonic acid, H${}_{2}$O${}_{2}$—oxygen peroxide.

Light emission by living organisms commonly known as bioluminescence is widespread phenomenon especially among deep-see species [16,17]. The enzymatic system responsible for light emission in luminous bacteria is one of the longest studied and one of the most complex [18,19]. Many aspects of its functioning under in vivo conditions and its biological role are still under consideration [16,20,21]. Bacterial luciferase, a key enzyme in the bioluminescent system, is a flavin-dependent monooxygenase utilizing reduced flavin mononucleotide, long–chain aldehyde, and oxygen as substrates [22]. In addition to the formation of highly energetic species in an electronically excited state, several other specific features distinguish this reaction from the reactions with studied diffusion control. First of all, it is a complicated multi-step process, including the formation of at least four reaction intermediates through the binding of three substrates (Figure 1). Due to the instability of one of them (reduced flavin mononucleotide), the complex non-steady state kinetics of light emission can be observed, which reflects the change of the rate-limiting stage during the reaction time course [22].

Secondly, the main “light” reaction pathway both in bacteria and in solution is accompanied by the numerous “dark” processes, the rates of which influence the light production efficiency of one luciferase molecule (Figure 1). It was shown that during the conventional in vitro assay, the reaction of not more than 16% of luciferase molecules results in the emission of light quantum [23]. This efficiency is relatively low in comparison with the bioluminescent reactions from other organisms (fireflies, coelenterates, etc.). This raises an interesting question of whether the rates of “light” and “dark” pathways are affected in a different way by intracellular conditions leading to the enhanced efficiency of the bioluminescent reaction in vivo.

The aim of the current work was to analyze the media viscosity effects on the individual rate constants of the stages of the bacterial bioluminescent reaction described above. On the one hand, this study was supposed to shed light on the physical and chemical mechanisms of regulation of bioluminescence function inside the bacterial cells. On the other hand, bacterial bioluminescence in vitro is widely used in clinical analysis, research of gene expression/regulation, environmental monitoring and other fields [24,25,26], therefore revealing ways to control bacterial luciferase activity is of great practical interest as well.

## 2. Materials and Methods

### 2.1. Materials

Flavin mononucleotide (FMN) was purchased from Sigma-Aldrich. EDTA (ROTH) was used as an electron donor for FMN photoreduction. Lyophilized recombinant luciferase *Photobacterium leiognathi* (99% purity) was purchased from Biolumdiagnostika Ltd. (Krasnoyarsk, Russia). Reactants were dissolved in a potassium phosphate buffer (0.05 M, pH 6.9). The concentrations of FMN and luciferase were determined spectrophotometrically with the extinction coefficients $\varepsilon_{445}=12.200$ M${}^{-1}$ cm${}^{-1}$ and $\varepsilon_{280}=80.000$ M${}^{-1}$ cm${}^{-1}$, correspondingly. The stock solution of decanal (Acros Organics, Fair Lawn, NJ, USA) in ethanol with 2·10${}^{-3}$ M concentration was used freshly prepared. For viscous media, we used mixtures of a buffer with glycerol (Panreac, Barcelona, Spain), sucrose (Gerbu, Heidelberg, Germany), sorbitol (Panreac), and glucose ( Reachim, Moscow, Russia) with a mass fraction of cosolvent of 10–40%.

### 2.2. Experimental Procedures

The kinetics of the reaction catalyzed by bacterial luciferase was recorded in a single-turnover assay [27]. Rapid autoxidation of reduced flavin after mixing with an air-equilibrated solution of luciferase makes a multiple enzyme turnover impossible and leads to flash-like bioluminescence kinetics. The bioluminescent reaction was initiated by mixing two solutions, A and B, using stopped-flow spectrometer SX20 (Applied Photophysics, Leatherhead, UK). To prepare Solution A, the buffer containing 3·10${}^{-5}$ M FMN and 1·10${}^{-2}$ M EDTA was made anaerobic by bubbling with argon for 10 min, and then a small volume of decanal solution was added. Solution B was an air-equilibrated buffer containing 1.9·10${}^{-6}$ M of bacterial luciferase. Before starting the reaction, FMN was photoreduced by exposure of the Solution A under the light of an incandescent lamp for 10 min. For experiments in viscous media, the mixtures of the buffer with glycerol or sucrose (10, 20, 30, 40 (*w/w*)%) were used as a solvent. The kinetics of bioluminescence was recorded under temperature control (at 20 ${}^{\circ }$C) for 15 s with a photomultiplier directly placed on the observation cell of the spectrometer without additional filters. Each kinetic curve was the averaging of at least 5 replicates.

The kinetics of *Intermediate I* formation was obtained by recording the absorbance change at 380 nm after mixing an anaerobic solution of luciferase (5 μM) and reduced flavin (30 μM) with the air-equilibrated buffer. This procedure was described elsewhere [22].

### 2.3. Mathematical Modelling of the Reaction Kinetics

The set of ordinary differential equations (Figure S1B) corresponding to the reaction scheme (Figure 1) was numerically solved by the program developed in Scilab software at the Laboratory of theoretical biophysics, the Institute of Biophysics SB RAS (Krasnoyarsk, Russia). The calculations resulted in the set of rate constants $k_{1}$, $k_{2}$, $k_{3}$, $k_{-3}$, and $k_{4}$, and theoretical kinetic curves. With a minimization parameter based on the least-squares fit error, the program searched for the best values of intensity at each time step that most accurately matched the experimental data (Figure S1C,D). Two decay constants ($k_{d}$, $k_{\mathrm{dd}}$) were fixed during simulation because they were determined in particular experiments [28]. Bioluminescence kinetic curves obtained at five different concentrations of decanal (10, 20, 30, 40 and 50 μM) in the buffer and in the presence of sucrose or glycerol were used as input data and simultaneously simulated. As an output, a set of individual kinetic constants was obtained for each medium. The relative error of each simulation did not exceed 3.8% (Figure S1E,F).

### 2.4. Data Analysis

From bioluminescence kinetics curves four parameters were calculated: the peak intensity (*I*${}_{max}$) as a maximum signal, the total quantum yield (Q*) as an area under the kinetic curve, the initial velocity of the reaction (*v*${}_{0}$) as a slope of the starting linear part of the kinetic curve, and the decay constant (*k*${}_{decay}$) as an indicator in the approximation of the final part of the kinetic curve by an exponential function. The details on calculating these parameters are given in the Supplementary Material (Figure S2).

The rate of *Intermediate II* formation, $k_{2}$, was determined by fitting absorbance change at 380 nm with the function $D_{380}=A\cdot e^{-k_{2}t}+B$ using Origin 8.0 software (OriginLab).

The dependences of the rate constants on the solution viscosity were fitted by a power function using Origin 8.0 software (OriginLab).

### 2.5. Computational Analysis of Bacterial Luciferase—Cosolvents Interactions

The results of three independent molecular dynamics (MD) runs performed for the crystal structure of *Vibrio harveyi* bacterial luciferase (PDB ID: 3FGC) [29] were used for the analysis. The structure was surrounded by water molecules and mixtures with glycerol or sucrose molecules adjusted to simulate the media with 10, 20, 30, 40 (*w/w*)% of cosolvent (Table S1). The details of MD simulation can be found in [30] and in the Supplementary Material. CASTp web-service was used to determine flavin and aldehyde binding cavity within the crystal structure of *V. harveyi* luciferase [31]. Then the side chains conformations of the selected residues were analyzed using the Bio3D module in R with a script developed by Haddad et al. [32,33,34]. In particular, for each residue the side chain dihedral angles were identified at each time step of MD trajectory and the corresponding conformation was assigned according to the rotamer library.The resulting data set included information on all conformations that were formed in the course of three independent MD calculations. Also the geometrical criterion was applied to define hydrogen bonds between the functionally important amino acids and cosolvent molecules. The H-bond was supposed to exist if the donor-acceptor distance is ≤3.5 Å and the hydrogen-donor-acceptor angle is ≤30${}^{\circ }$.

## 3. Results

### 3.1. Kinetic Effects of Media with Glycerol and Sucrose on the Bioluminescent Reaction Catalyzed by Bacterial Luciferase

**Figure 2:**
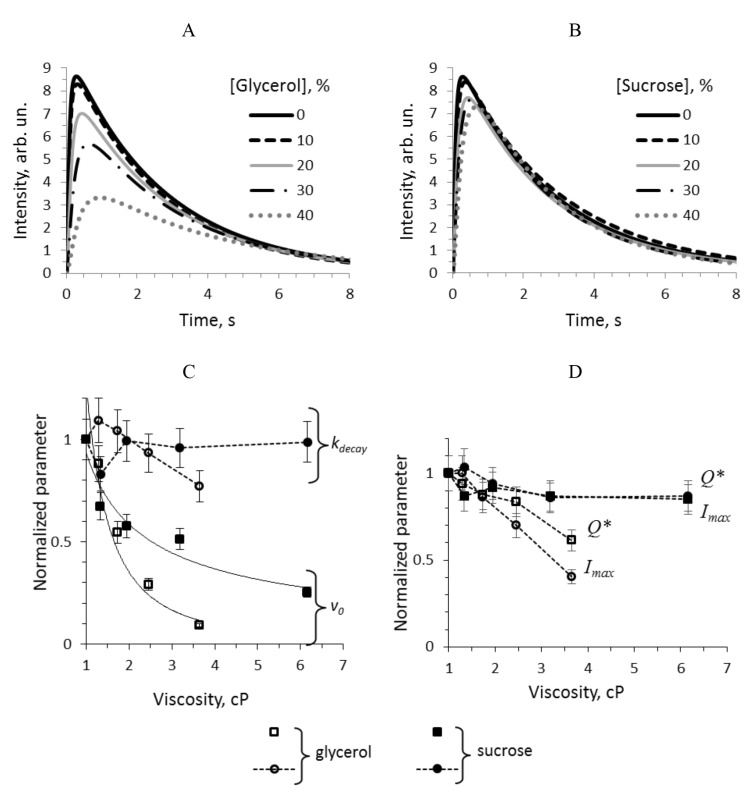
The kinetic curves of the bioluminescent reaction with 40 μM of decanal in media with 10, 20, 30 and 40% of glycerol (**A**) and sucrose (**B**) and dependence of their characteristics on the media viscosity: the decay constant and initial velocity (**C**); the peak intensity and the total quantum yield (**D**). The parameters were normalized in accordance with the values in the buffer (viscosity of 1 cP). The solid lines are the power law approximation with the exponent −0.67 for sucrose solutions and −1.85 for glycerol ones. The dashed lines are shown to guide the eyes.

The kinetics of light emission from the reaction of about 1 μM bacterial luciferase with 15 μM of reduced flavin mononucleotide and 10–50 μM of decanal was recorded in media with different concentration of glycerol and sucrose using stopped-flow technique. During 8–10 s after mixing reduced flavin with aldehyde and luciferase, the bioluminescence intensity rises rapidly, reaches a maximum, and slowly decreases (Figure 2A,B), which is the consequence of the fact that reduced flavin is autoxidized within 0.5 s, and luciferase can make only single turnover. Four empirical parameters can describe such kinetic curve: the peak intensity (*I*${}_{max}$), the decay constant (*k*${}_{decay}$), the total quantum yield (Q*), and the initial velocity of the reaction (*v*${}_{0}$). The dependence of these characteristics on media viscosity in the reaction with 40 μM of decanal is presented in Figure 2C,D. One can notice that: (i) the kinetic effects of two viscogenic agents are different, (ii) among all the parameters only the initial velocity *v*${}_{0}$ demonstrates the power-low dependence on viscosity, and (iii) higher concentration of glycerol (30 and 40%) causes the decline of all the characteristics, which is different from the sucrose solutions effect.

Similar data obtained for other aldehyde concentrations can be found in the Supplementary Material (Figure S3) as well as the dependence of the characteristics on the media viscosity (Figure S4). It was revealed that for 20, 30 and 50 μM of decanal, the kinetic parameters change in the same manner as shown in Figure 2C,D, and only for the lowest aldehyde concentration (10 μM) the patterns can be different.

These empirical parameters are widely used to describe the kinetics of the bacterial luciferase reaction because they are easy to determine. In general, each of them is the result of some combination of the individual rates of reaction steps. To understand the molecular mechanisms of the media effects underlying the found kinetic changes, we aimed to retrieve the rates of the separate reaction stages from the kinetic curves using mathematical modeling techniques and particular experiments.

### 3.2. Mathematical Modeling of the Kinetics of the Bacterial Bioluminescent Reaction

The kinetic mechanism of the bacterial bioluminescent reaction has been studied intensively for several decades, and, as a result, some comprehensive kinetic models have been proposed [22,35]. In Figure 1 and Figure S1A, the basic reaction stages included in almost all the proposed models are presented. The first of them is reduced flavin, FMNH${}_{2}$, binding by luciferase with the formation of enzyme-substrate complex E·FMNH${}_{2}$ (*Intermediate I*). This complex reacts with the molecular oxygen to form C(4a)-hydroperoxyflavin, E·FMNHOOH (*Intermediate II*). Then it interacts with the aldehyde via a nucleophilic attack to produce C(4a)-peroxyflavin hemiacetal, E·FMNOOH·RCOH, (*Intermediate IIA*), which decomposes into a carboxylic acid and electronically excited intermediate C(4a)-hydroxyflavin associated with luciferase, E·FMNOH [22]. Deactivation of the last species leads to emission of light in the range 460–530 nm. In the absence of aldehyde, C(4a)-hydroperoxyflavin is protonated and decomposes to H${}_{2}$O${}_{2}$ and FMN through the dark pathway.

Basing on this reaction scheme, we created the system of differential equations establishing the relations among the concentrations of reaction substrates, the intermediates, and the rate constants of their interconversion (see Supplementary Material, Figure S1B). This model was used to fit the experimental kinetic curves with 7 rate constants: $k_{d}$, $k_{\mathrm{dd}}$, $k_{1}$, $k_{2}$, $k_{3}$, $k_{-3}$, $k_{4}$. Two of them ($k_{d}$, $k_{\mathrm{dd}}$) were preliminarily estimated for each viscous solution in the particular experiments as it is described in [28]. Therefore, they were fixed during the modeling sessions.

**Figure 3:**
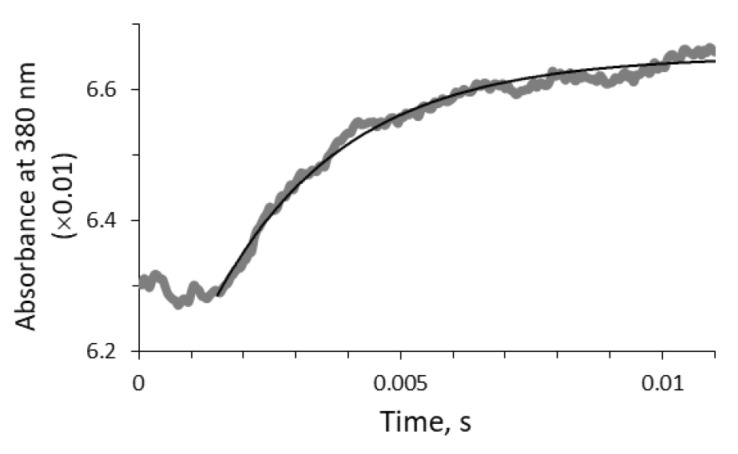
The absorbance change during C(4a)-hydroperoxyflavin intermediate formation in the buffer providing the rate constant $k_{2}$. The gray thick line refers to the experimental kinetic curve, the black thin line—to the fitting curve with function $D_{380}=A\cdot e^{-k_{2}t}+B$.

The third rate constant, $k_{2}$, of the C(4a)-hydroperoxyflavin intermediate formation was determined by analyzing the absorbance changes at 380 nm after mixing the air-equilibrated buffer with an anaerobic solution containing luciferase and reduced flavin (Figure 3). This reaction proceeded quickly (within 15 ms) and the corresponding rate constant was estimated as $k_{2}$ = 400 s${}^{-1}$. It turned out to be impossible to experimentally determine the $k_{2}$ value for the solutions of glycerol or sucrose due to strong distortion of the optical signal by the cosolvents within 50 ms after mixing. The found constant of 400 s${}^{-1}$ in the buffer was used as the initial $k_{2}$ value for kinetics simulations in viscous media.

**Figure 4:**
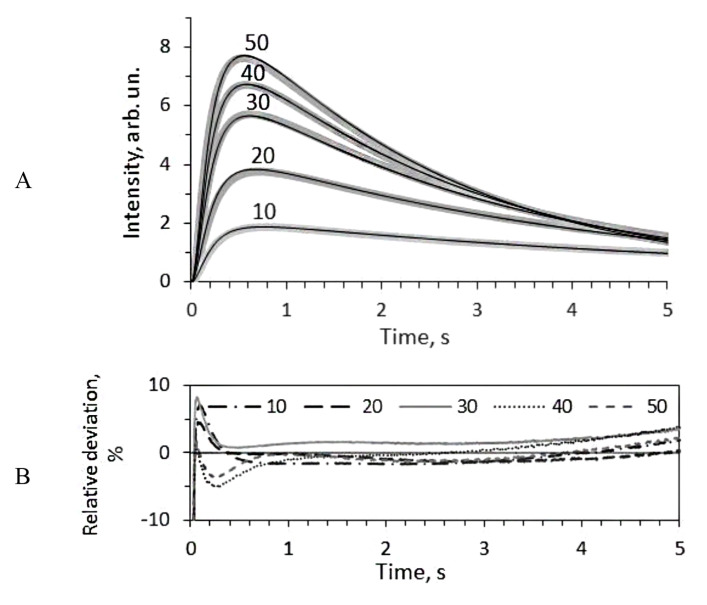
The kinetic curves of the bioluminescent reaction with 10, 20, 30, 40 and 50 μM of decanal in media with 30% of sucrose (the gray thick lines), their simulation by mathematical model with the constants indicated in Table 1 (the black solid lines) (**A**), and corresponding relative deviations of the simulated curves from the experimental ones (**B**).

To find the rest of the rate constants the set of kinetic curves of the bioluminescent reaction was modeled using the program adjusting $k_{1}$, $k_{2}$, $k_{3}$, $k_{-3}$, and $k_{4}$ to satisfy all the curves. Each set included five kinetic curves recorded with different concentrations of decanal (10, 20, 30, 40 and 50 μM). The example of experimental and simulated kinetic curves is shown in Figure 4. The found rate constants for each media are collected in Table 1.

From the Figure 4B, one can see that the modeled curves deviate from the experimental ones by not more than 5% in the major part of the reaction time course (0.2–5 s). Thus, the developed model satisfactorily describes the kinetics of the bacterial bioluminescent reaction under non-steady-state conditions.

Nevertheless, one important remark should be made about the irreversibility of the first reaction stage on the scheme in Figure 1. It is known that *Intermediate I* can dissociate into an enzyme and a reduced flavin mononucleotide, and for *V. harveyi* luciferase, the rate constant of this process was estimated as 1200 s${}^{-1}$ [22]. In our preliminary simulations by the model, including the reverse process of the first stage, it was found that the rate constant for flavin dissociation could vary from 300 to 2000 s${}^{-1}$ with no influence on the other rate constants and fitting quality, both in the buffer and in viscous media. The probable reason is the high rate of the next reaction stage ($k_{2}$). Thus, we excluded *Intermediate I* dissociation rate constant from the final kinetic model to facilitate the calculation.

### 3.3. Dependence of the Individual Rate Constants on Media Viscosity

**Figure 5:**
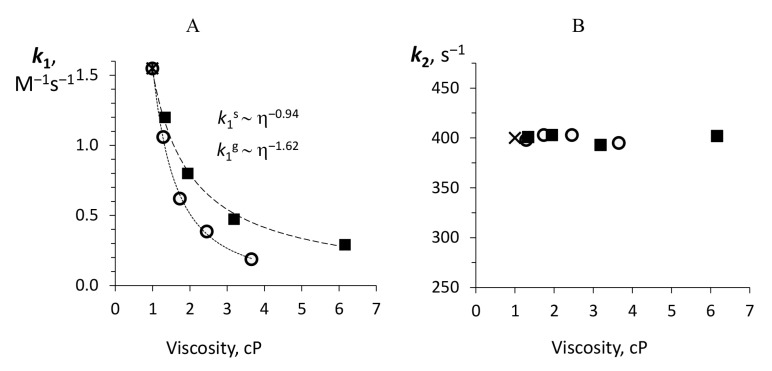
The dependence of the rate constants for the reduced flavin binding, $k_{1}$ (**A**) and C(4a)-hydroperoxyflavin formation, $k_{2}$ (**B**) on viscosity for glycerol (the empty markers) and sucrose (the filled markers) solutions. The dashed lines refer to the power law approximation with the indicated exponents. The cross shows the value in the buffer solution. Superscripts *g* and *s* refer to glycerol and sucrose correspondingly.

The rate constants of reduced flavin binding by luciferase, ($k_{1}$), in media with glycerol and sucrose are shown in Figure 5A. One can see that in sucrose solution, it behaves like a diffusion-controlled process: with increasing viscosity, it decreases as a power-law function with index −0.94. However, in the case of glycerol solutions, the enhanced effect is observed: the exponent value is about −1.62.

The effect of sucrose and glycerol solutions on the rate of formation of C(4a)-hydroperoxyflavin, adjusted by the model from the initial value of 400 s${}^{-1}$, is shown in Figure 5B. The obtained data indicate that this stage of the reaction is not sensitive to diffusion. Previously, it was found that the formation of C(4a)-hydroperoxyflavin occurs much more slowly when a luciferase enzyme is mixed with free FMNH${}^{-}$ in an air-saturated solution, which means that the binding of free FMNH${}^{-}$ to luciferase limits the overall reaction [36,37]. Thus, it is impossible to draw an unambiguous conclusion about the influence of sucrose and glycerol on ($k_{2}$), since this is not a rate-limiting stage.

**Figure 6:**
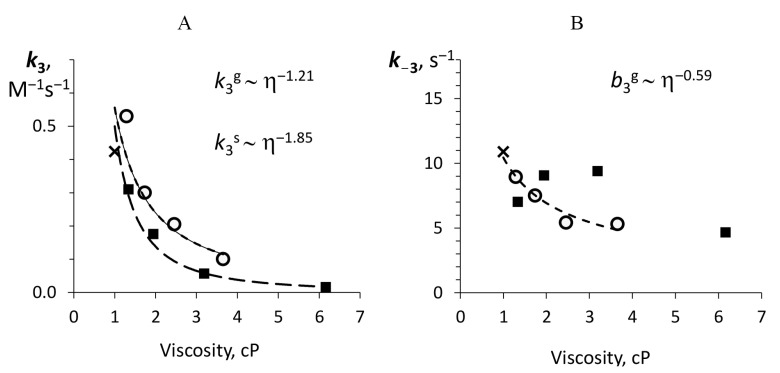
The dependence of the rate constants of decanal binding (**A**) and dissociation (**B**) on viscosity for glycerol (empty markers) and sucrose (filled markers) solutions. The dashed lines refer to the power law approximation with the indicated exponents. The cross shows the value in the buffer solution. Superscripts *g* and *s* refer to glycerol and sucrose correspondingly.

The strong effect of viscosity is observed on the binding of aldehyde (*Intermediate IIA* formation), but not on its reverse dissociation (*Intermediate IIA* decay), as shown in Figure 6. The power-law dependence of aldehyde binding, $k_{3}$, on viscosity is characterized by the indices −1.86 and −1.21, which means that the other factors enhance the significant diffusional limitation (e.g., cosolvent interaction with the protein and substrate and/or internal protein dynamics).

**Figure 7:**
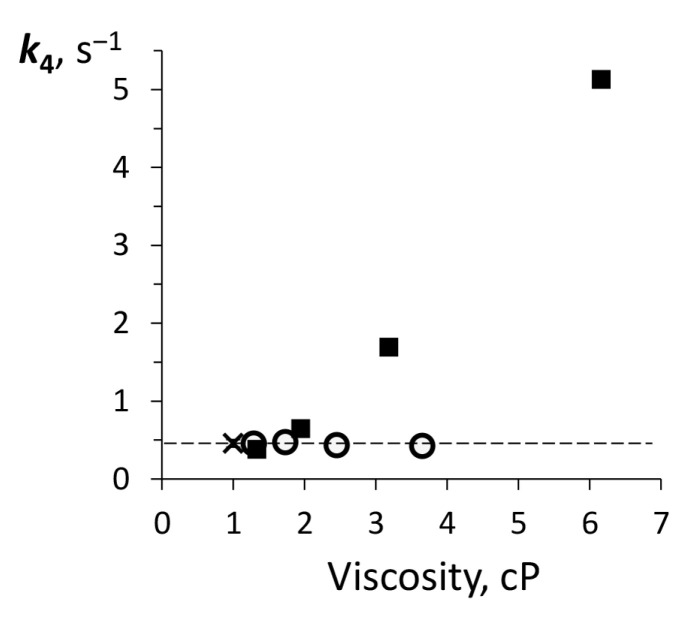
The dependence of catalytic constant of bacterial luciferase ($k_{4}$) on viscosity for glycerol (empty markers) and sucrose (filled markers) solutions. The dashed line refers to the value in the buffer solution marked with a cross.

The final chemical stage of the reaction catalyzed by bacterial luciferase is the formation and deactivation of electronically excited species which is supposed to be enzyme-bound C(4a)-hydroxyflavin ($k_{4}$ in Figure 1) [37]. The rate of this step can be regarded as a measure of the enzyme catalytic activity because it reflects the ability of luciferase to produce light as a product. The dependence of $k_{4}$ on media viscosity is shown in Figure 7.

One can see that the effects of two viscogenic agents on $k_{4}$ are different: sucrose enhances this rate, while in the presence of glycerol it remains the same as in the buffer.

Thus, the analysis of the kinetic curves with the mathematical model revealed that the substrate binding is a diffusion-controlled stage of the luciferase reaction ($k_{1}$, $k_{3}$), whereas the catalytic constant of the reaction ($k_{4}$) is not. To identify the specific influence of glycerol and sucrose on the structure of bacterial luciferase that can underlie observed effects the molecular dynamics methods were applied.

### 3.4. Effects of Glycerol and Sucrose Molecules on Bacterial Luciferase Structure

The molecular dynamics of *V. harveyi* luciferase surrounded by water molecules or mixtures of water with sucrose/glycerol was simulated in three independent runs of 40 ns each [30]. In the current study, we analyzed local structural and dynamic properties of the protein that alter in the presence of the cosolvents. Namely, the sampling of amino acid conformations of selected residues in the luciferase active site was performed using the previously obtained MD trajectories. It allowed revealing the intrinsic conformational preferences induced by protein-cosolvent interactions. Additionally, we studied if the residues involved in the substrates binding participate in hydrogen bond formation with the cosolvents. It is important to mention that all residues discussed below are identical for 21 luciferase sequences of different luminous species, including *V. harveyi* and *P. leiognathi* (Figure S5).

**Figure 8:**
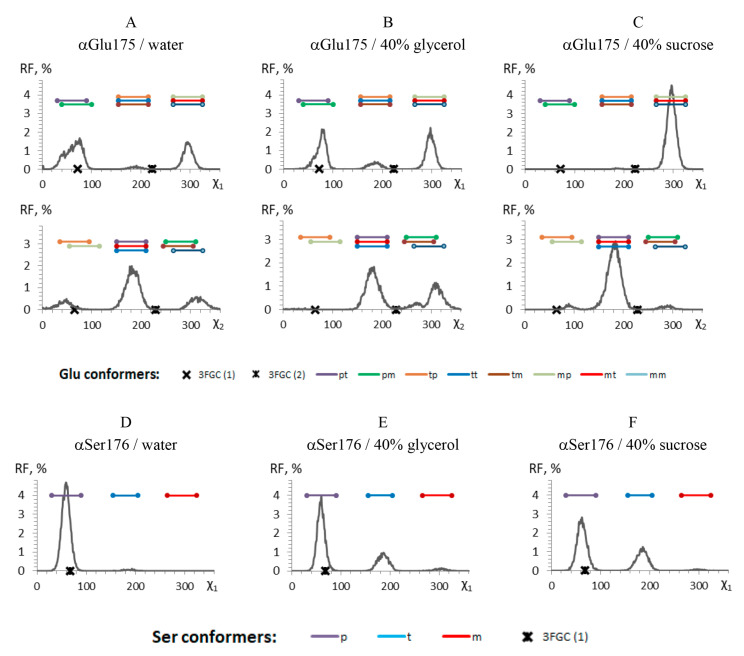
The relative frequency (RF) of side-chain conformations of the selected residuals during the molecular dynamic simulation in water and high concentration of the cosolvents: (**A**–**C**)—$\chi_{1}$ (the upper panel) and $\chi_{2}$ (the lower panel) dihedrals of αGlu175; (**D**–**F**)—$\chi_{1}$ dihedral of αSer176. The colored straight lines indicate the dihedral intervals of the known conformations (according to [39]). The crosses indicate the conformers found in the luciferase crystal (PDB ID: 3FGC) [29].

The influence of glycerol or sucrose molecules on the side chain dihedrals of the amino acid residuals known to be important for the efficiency of the luciferase reaction, [38] was studied. It was found that among six residues, which are involved in the binding of flavin (Figure S5) (αGlu43, αArg107, αLeu109, αThr179, αGlu175, αSer176), the latter two change their preferred conformation in the presence of the cosolvents. Figure 8C demonstrates that in 40% of sucrose αGlu175 takes the only conformation, designated as **mt**, unlike the cases in water or 40% of glycerol (Figure 8A,B). The αSer176 in the presence of cosolvents was found to has two main conformations (**t** and **p**), while in water, it takes preferably only **p** conformation (Figure 8D–F). The data for the other cosolvent concentrations can be found in Figure S6.

Moreover, we revealed that the change of the αGlu175 and αSer176 conformation is accompanied by an increase in their solvent accessible surface area (SASA) (Figure S7). This effect is more pronounced for sucrose solutions (Figure S7B) and not only for the two mentioned amino acids, but also for the αArg125 and αArg290 as well. These arginines are known to play an important role in luciferase mobile loop functioning [40]. The SASA of each amino acid was calculated as the mean over the time of the protein molecular dynamics simulation, and large standard deviations shown in Figure S7 can be a result of amino acid mobility.

The study of the conformations of amino acids which are supposed to interact with the aldehyde in the active site of luciferase [41] (αPhe49, αTyr110, αTrp194, αIle195, αTrp250, αTyr251 and αTyr254) (Figure S5) during molecular dynamics in the presence of sucrose or glycerol molecules detected no change caused by the cosolvents.

Additionally, the H-bonding between the protein atoms involved in the binding of flavin (which are nitrogens of αGlu43, αArg107, αLeu109, αGlu175, αSer176 and oxygens of αSer176, αThr179) and atoms of the cosolvents were analyzed. It was found that hydrogen bonds fractional occupancy is near zero for the majority of the atoms (Figure S8). i.e., they form no H-bonds with glycerol or sucrose. It is notable, that for nitrogens of αArg107 and oxygen of αSer176, the highest occupancy of about 15% was revealed, and that can only be a marker of the unstable H-bonds between these atoms and cosolvent molecules (Figure S8B,C,G). Similar analysis of interaction of the aldehyde binding site with the glycerol or sucrose molecules revealed that all tryptophan and tyrosine residues can form hydrogen bonds with glycerol and only αTyr251 and αTyr254—with sucrose. The highest occupancy among all studied residues was found for αTrp194 (80%) indicating the possibility of a stable H-bond between its side chain nitrogen and glycerol.

**Figure 9:**
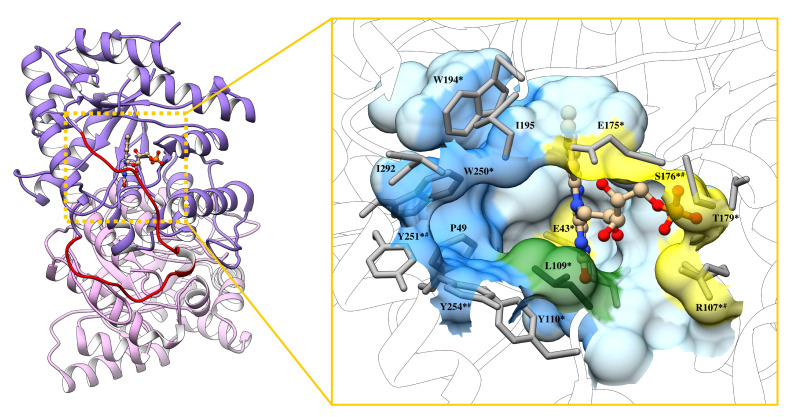
The crystal structure of *V. harveyi* luciferase (PDB ID: 3FGC) in complex with FMN (CPK representation) in the active site. The mobile loop segment is indicated in red, α and β–subunits—in purple and pink, correspondingly. The surface of luciferase active site cavity was determined by CASTp [31]. Yellow area—amino acid residues that stabilize FMN; blue—the hydrophobic cavity for aldehyde [29,41]. The αLeu109 is involved in the binding of both substrates (green). The superscripts indicate the presence of hydrogen bonds with glycerol (*) and with sucrose (${}^{\#}$) during MD simulation time (Figures S8 and S9). No superscript means that the H-bonds were not found.

The relative position of the luciferase residues that form the active site of the enzyme is shown in the Figure 9. Amino acids for which formation of stable or unstable hydrogen bonds with the cosolvents was observed are marked by the superscripts. H-bonding between cosolvent molecules and substrate binding residues indirectly reflects the extent of penetration of the glycerol/sucrose molecules into the cavity where active site is located (Figure 9). Therefore, glycerol definitely penetrates deep inside the catalytic gorge, because it interacts with the αGlu43. To check if sucrose molecules can approach this residue without forming of hydrogen bond, the minimal distance between any atoms of αGlu43 and cosolvent molecules during molecular dynamic simulations was estimated using GROMACS software [42]. In models with 40% of cosolvents the distance of 1.72 ± 0.20 Å was observed for glycerol and 9.15 ± 0.59 Å—for sucrose. For water molecules the distance of 1.45 ± 0.10 Å was obtained. This result confirms that the glycerol molecules can penetrate deeper into the catalytic gorge of the luciferase than sucrose ones.

### 3.5. The Dependence of Luciferase Kinetics on the Molecular Size of Viscogenic Cosolvent

**Figure 10:**
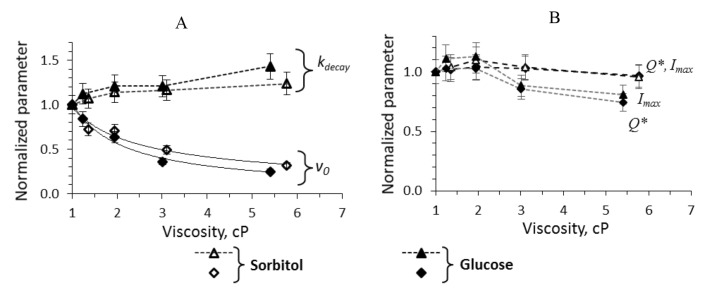
Dependence of the kinetic characteristics of luciferase reaction in the presence of glucose (solid markers) and sorbitol (empty markers) on solution viscosity: decay constant and initial velocity (**A**); peak intensity and total quantum yield (**B**). The solid lines are the power law approximations with the exponent of −0.85 for glucose solutions and −0.62 for sorbitol solutions. The dashed lines are shown to guide the eyes. A decanal concentration in the reaction was 50 μM. The viscosity values of the solutions were taken from [43,44] for glucose and sorbitol, respectively.

Thus, summarizing all the obtained results, we propose that the important mechanism responsible for affecting luciferase activity by the cosolvents is penetration of their molecules into the active center of the enzyme. To check this hypothesis, we measured the kinetics of luciferase reaction in the presence of 10–40% of cosolvents with similar molecular sizes but different chemical nature. We chose sorbitol, which is about 2-fold larger than glycerol, and glucose, which is about 2-fold smaller than sucrose. The results of the test experiments are given in Figure 10.

As it can be seen, the effects of sorbitol and glucose are very similar to a sucrose’s one (compare Figure 2C,D and Figure 10). Both cosolvents change the initial velocity $v_{0}$ in a diffusion-controlled manner and tend to slightly increase the decay constant value (Figure 10A). The similarity of the effects obtained for sorbitol and glucose correlates with their close size: the effective hydrodynamic radii of these molecules were reported as 3.9 and 3.6 Å, correspondingly [45]. Those of sucrose and glycerol are 5.2 and 3.1 Å. It is worth noting that ethylene glycol which is smaller than glycerol (effective hydrodynamic radii is about 2.6 Å [46]) appeared to decrease the total quantum yield of the bacterial bioluminescent reaction in vitro [47]. Thus, the test experiments support the assumption about critical role of the molecular size of viscogenic cosolvent in its influence on the reaction catalyzed by bacterial luciferase.

## 4. Discussion

This work aimed to elucidate the impact of media viscosity on bacterial luciferase functioning. The non-steady-state kinetics of the reaction catalyzed by *P. leiognathi* bacterial luciferase in the presence of various concentrations of sucrose and glycerol was studied using the stopped flow technique. The obtained data show that additionally to the general slowing down of the reaction kinetics (which can be the result of diffusion control of some reaction stages), there are specific effects of the media on the catalytic activity of the enzyme. The different influence of the cosolvents is reflected in a dependence of empirical parameters of bioluminescent reaction ($I_{max}$, $k_{decay}$, Q*, $v_{0}$) on viscosity (Figure 2). Increasing the concentration of glycerol solutions, a gradual decrease in the peak intensity ($I_{max}$) and luminescence decay rate ($k_{decay}$) was observed; these parameters, however, remained approximately the same in the presence of sucrose (Figure 2). The total quantum yield (Q*) of the luciferase reaction is assumed to be linearly proportional to the peak intensity ($I_{max}$) and inverse decay constant (1/$k_{decay}$) [48], but in the case of glycerol solutions, it correlates with the first parameter rather than the last. As it has been shown previously, the luminescence decay constant ($k_{decay}$) depends on the rate of dark decay of the *Intermediate II* ($k_{dd}$), the catalytic constant ($k_{4}$), aldehyde binding velocity (*k*${}_{3}$, *k*${}_{-3}$), and the aldehyde concentration (Figure S2B) [48]. Our data indicate that the effects of glycerol and sucrose on the rate(s) of some of these elementary reaction steps are different, which was supported by our further results of kinetics modeling.

To reveal the mechanisms underlying the observed effects, we developed a mathematical model of the reaction kinetics and recovered the individual rate constants of the reaction steps from the sets of experimental kinetic curves. We proposed a kinetic model of reaction catalyzed by *P. leiognathi* luciferase (Figure 1) using a similar approach, as it was done previously for *V. harveyi* luciferase [22]. Despite a resembling reaction mechanism, these two luciferases are different in emission decay rates and designated as “fast” (*P. leiognathi*) and “slow” (*V. harveyi*) enzymes [49]. Distinctive features of our model are: (i) irreversibility of reduced flavin binding by luciferase and elimination of the next enzyme isomerization step; (ii) combining of the several steps after aldehyde binding into one final stage with the rate constant *k*${}_{4}$. Nevertheless, this simplified model with seven rate constants (two of which, $k_{d}$ and $k_{dd}$, were determined in particular experiments and fixed during simulation) allowed us to fit the experimental kinetic curves with the relative error <3.8% and to obtain the dependence of the rates of the main reaction stages on media viscosity. The expanding of the model with the additional stages did not improve the quality of kinetic curves fitting. We obtained the dependences of the rate constants ($k_{1}$, $k_{2}$, $k_{3}$, $k_{-3}$, $k_{4}$) on the media viscosity using a kinetic model. Similar to the decay reaction steps with $k_{d}$ and $k_{\mathrm{dd}}$ rate constants, the substrates binding reactions ($k_{1}$, $k_{3}$) proceed in a diffusion-controlled manner. It is supported by the fact that the dependence of these rate constants on media viscosity corresponds to a power function with an exponent, close to 1 and higher (Table 1).

It was found that flavin binding ($k_{1}$) is a clearly diffusion-controlled process in the presence of sucrose, while in glycerol solutions enhanced effect was observed (δ > 1). The latter can be explained by the findings from molecular dynamics that glycerol, unlike sucrose, can penetrate the active site and form hydrogen bonds with αGlu43 responsible for flavin binding and located at the bottom of the catalytic gorge (Figure 9).

The aldehyde binding stage ($k_{3}$) turned out to be heavily influenced by viscosity both in glycerol and in sucrose solutions (Figure 6A) (with δ of 1.21 and 1.85). This effect can also be attributed to involvement of some functional residues in H-bonding with glycerol (especially αTrp194), but one can suggest other mechanisms of substrate-cosolvent and protein-cosolvent interactions. For example, hydrogen bonding between aldehyde substrate and cosolvent molecules can lead to an increase in the effective hydrodynamic radius that aggravates diffusion hindrance. It is worth noting that the solutions used with higher viscosity also have a lower polarity in comparison with the buffer. In a less polar environment, the substrates can change their preferential conformation that will affect the diffusion rate. This is especially relevant for aliphatic aldehyde, which is a highly hydrophobic compound. The cosolvent molecules can also interfere with enzymatic activity by blocking the access of aldehyde to the active site. Another possible mechanism of the enhanced viscosity effect is an influence on internal relaxation of luciferase intermediates, which contribution to overall reaction rate is negligible in buffer, but can become significant in the presence of glycerol and sucrose. In this case, the protein becomes dynamically sluggish, so the complex formation with substrates is lagged [50]. The dependence of the reverse aldehyde dissociation ($k_{-3}$) on viscosity does not reflect a diffusion control, though a slight effect can be observed for glycerol solutions. There are examples when diffusion controls the release of the substrate from the active site [51], but it seems that it is not the case here.

No dependence on solution viscosity was found for the stage of the oxygen binding by the luciferase-flavin complex ($k_{2}$) (Figure 5B); $k_{2}$ in the buffer was determined experimentally as 400 s${}^{-1}$ and used as an initial value for kinetics simulation in viscous media. The obtained $k_{2}$ is close to the value published earlier for *V. harveyi* luciferase reaction in an air-equilibrated buffer [22]. In [22], the authors showed that the rate of oxygen binding by the luciferase-flavin complex depends on oxygen concentration, so it is a second-order process. However, in our work we use a pseudo-first-order value, since all the experiments were conducted under high oxygen concentrations (about 120 μM) compared to the concentrations of the other reactants (0.9–50 μM). An additional check of the dissolved oxygen level in the used glycerol and sucrose solutions confirmed that it is close to its concentration in the buffer. The lack of diffusion limitation for $k_{2}$ obtained in our work can be a result of the high affinity of bacterial luciferase to oxygen [22,35], so that $k_{2}$ does not govern the non-steady-state reaction kinetics neither during the first (rise) nor during the last (decay) phase.

The most remarkable difference between glycerol and sucrose effects on the reaction catalyzed by bacterial luciferase was found in a variation of the rate of electronically excited intermediate formation, $k_{4}$ (Figure 7). Sucrose clearly promotes an increase of $k_{4}$, while glycerol has no effect. Although many cosolvents are known as stabilizers, the cases of activation of the enzymatic reaction by them are rare. The increase in the rate constant of enzymatic catalysis caused by cosolvents can be explained by different mechanisms, for example, a shift of the dielectric constant of media in the presence of glycerol or sucrose [52], or participation of nucleophilic cosolvent as a reactant in a reaction of hydrolytic enzymes [53]. For reaction catalyzed by butyrylcholinesterase in solution with sucrose it was deduced that sucrose is excluded from the active site cavity and acts as a semi-permeable membrane at its entrance. This protein-cosolvent interaction induces osmotic stress that leads to a transfer of water molecules from the cavity to the bulk solution [12]. This can also be the case for the interaction of sucrose with bacterial luciferase because our molecular dynamics simulations revealed that sucrose cannot enter its catalytic gorge. The hydration change in the active site of luciferase may cause hydrogen bonds rearrangement, which is critical for *Intermediate IIA* stabilization [54].

A comparison of changes in empirical parameters of the reaction kinetics ($v_{0}$, $k_{decay}$, $I_{max}$ and Q*, Figure 2C,D) and in individual rate constants determined by separate experiments and the model ($k_{d}$, $k_{dd}$, $k_{1}$, $k_{3}$, $k_{-3}$ and $k_{4}$, Figure 5, Figure 6 and Figure 7) revealed some patterns. Firstly, apparent initial rate of the reaction ($v_{0}$) behaves in a diffusion-controlled manner close to the stage of flavin binding ($k_{1}$). Secondly, $k_{decay}$ change does not demonstrate any clear correlations with separate stages rates in viscous media; $k_{decay}$ is a function dependent on dark decay rate constant $k_{dd}$, aldehyde association $k_{3}$/$k_{-3}$ and catalytic constant $k_{4}$ [48], which, in their turn, are impacted by solution viscosity differently. Therefore, the change of empirical parameter $k_{decay}$ does not help to define which individual stage is affected by, because $k_{decay}$ value is not controlled by any one rate constant, but is the result of a complex non-linear combination of the rates of three reaction stages.

One of the general problems we addressed in our study is whether the increased viscosity of the media shifts the balance between “light” and “dark” pathways of the bioluminescent reaction catalyzed by bacterial luciferase. It is known that the efficiency of this reaction in a buffer is about 10–16% [38], i.e., only one or two of ten luciferase molecules produce a light quantum in a reaction. It indicates that the main reaction pathway is accompanied by the intense secondary (“dark”) processes which can be influenced by media viscosity at different extent. Our results showed that the presence of cosolvents makes the rate of “dark” processes ($k_{d}$, $k_{dd}$) slower, yet rate constants of substrate binding are also reduced ($k_{1}$, $k_{3}$). The relation between δ-exponents in viscosity dependencies for “light” and “dark” stages (Table 1) points out that the bioluminescence yield of the reaction does not profit from the simple slowing down of the components’ diffusion under the used conditions. The possible conclusion is that the main contribution to the reaction efficiency is made by catalytic constant $k_{4}$, and there are some mechanisms to increase this rate and to improve the efficiency of the light emission process in viscous media.

Thus, the analysis of bacterial luciferase functioning in solutions with glycerol and sucrose demonstrates the different (specific) action of these cosolvents on the enzyme catalytic constant $k_{4}$, in addition to the general effect of diffusional control of substrates binding. To elucidate the mechanisms of the found specific effects we used classical all-atom molecular dynamics simulation in explicit solvent, which is routinely applied to reveal the peculiarities of protein-cosolvent interaction [11]. Earlier we showed that no significant conformational changes of bacterial luciferase occur in solutions with 10–40% of sucrose or glycerol, which was confirmed by unchanged RMSD (root mean square deviation) and SASA (solvent accessible surface area) of the protein [30]. Additionally, it was found that the functionally important mobile loop of luciferase (262–291 a. r.) demonstrated higher mobility at low concentration of the cosolvents (≤10%). Also previously we estimated the amount of water and cosolvent molecules in the active site of bacterial luciferase as an average value by the last 20 ns of the molecular dynamics modeling [30] and revealed that it can contain glycerol molecules but not sucrose. In the current study, we analyzed conformational preferences of amino acid residues responsible for flavin and aldehyde binding, their involvement in H-bonding with cosolvent molecules and penetration of cosolvents into the active site gorge.

Thorough analysis of various structural parameters revealed two characteristics, which are different between the modelled systems containing glycerol or sucrose: (i) conformation of amino acid residues important for enzyme catalysis and (ii) minimal distance between αGlu43 located at the bottom of the active center gorge and cosolvent molecule.

**Figure 11:**
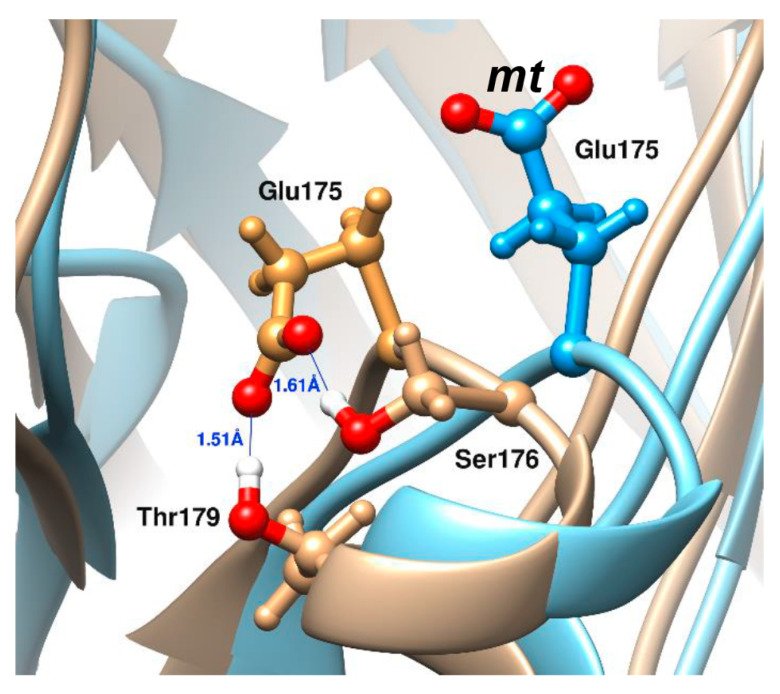
Two conformations of αGlu175 side chain in active site of bacterial luciferase. The superposition of luciferase structures taken from MD-simulations with no cosolvent (tan) and in presence 40% of sucrose (blue) is shown (the snapshots time is 30 ns). In water surrounding and in the presence of glycerol, αGlu175 can take a conformation stabilized by two strong H-bonds with αThr179 and αSer176 (side chains are shown as ball-and-sticks in tan color). In the presence of 20–40% of sucrose, the only conformation of αGlu175 side chain is one shown in blue color (**mt** conformation according to [30]). In red the oxygen atoms are depicted.

Functionally important residue αGlu175 of bacterial luciferase was found in two main conformations during the MD simulation (Figure 11). Both found conformations are close to those resolved in a protein crystal structure (crosses in Figure 8A–C) [29]. The first of them (shown in tan in Figure 11) is formed in water and in the presence of glycerol. It is stabilized by two strong H-bonds with αThr179 and αSer176, which prevents a proper binding of flavin in the enzyme active site (it was shown by a molecular docking simulation [55]). The second αGlu175 conformation (**mt**, shown in blue in Figure 11), which is more exposed to the solvent, corresponds to that detected in luciferase-flavin complex [29,55]. We found that it is partially formed in water surrounding and in the presence of glycerol; meanwhile, in the presence of sucrose we observed this αGlu175 conformation almost in all the cases (Figure 8A–C). Thus, our results indicate that sucrose solutions probably promote the adoption of an active conformation of the active center of bacterial luciferase. This effect can account for the dependence of the luciferase catalytic constant $k_{4}$ on the sucrose-mediated viscosity (Figure 7). It was shown that an αGlu175Gly mutation weakens aldehyde binding affinity and influences the other steps of the reaction [55].

The second important difference between sucrose and glycerol effects on luciferase concerns the accessibility of the enzyme active site for cosolvent molecules. While sucrose molecules can form H-bonds only with the residues located at the active site entrance, glycerol can penetrate to the deeper area of the gorge (Figure 9). It is also reflected in the hydrogen bonds occupancy between protein atoms, which participate in flavin or aldehyde binding, and cosolvent molecules (Figures S8 and S9).

In general, the different effects of glycerol and sucrose on enzymes functioning were demonstrated many times (for butyrylcholinesterase [12], fatty acid synthase [56], carboxypeptidase-A [57], and H${}^{+}$-ATPase [58]). The observed difference is assumed to be due to the molecular size of these cosolvents. Smaller glycerol molecules could penetrate closer to the catalytically important protein sites and probably block the groups responsible for the proper binding of the substrate, while sucrose is large enough to be excluded from the active site [12,59].

## 5. Conclusions

The principles of enzymes functioning under natural or in vivo-like conditions are of great importance for understanding the metabolic processes which support cellular bioenergetics, growth, and survival. Classical in vitro studies cannot reproduce a cell interior, which is highly heterogeneous, because it is composed of macromolecules (carbohydrates, lipids, proteins, and nucleic acids), osmolytes, electrolytes, metabolites, and water. This complexity of intracellular environment can suppress the diffusion of cell components and cause the reduction of biochemical reactions rate. However, in some cases, diffusional restrictions have a key functional significance. The purpose of this research was to identify the mechanisms underlying the influence of diffusion on the bioluminescent reaction catalyzed by bacterial luciferase. Glycerol and sucrose were used as low molecular weight viscogenic agents.

The identified changes in the reaction kinetics point out that, apart from solution viscosity, some additional characteristics of the cosolvent contribute to the media effect: in spite of slowing down of the light emission kinetics in the presence of the both cosolvents, glycerol caused pronounced reduction of peak intensity and overall quantum yield, while sucrose effects were weak. In order to unveil the mechanisms responsible for the observed difference, we analyzed the change of rate constants of separate reaction steps in accordance with the conventional kinetic scheme of bacterial bioluminescent reaction. For this purpose, we developed a mathematical model that describes the kinetics of light emission in time course of luciferase reaction.

The results of modelling showed that binding of reduced flavin (*Intermediate I* formation) and aldehyde (*Intermediate IIA* formation) occur in a diffusion-controlled manner, while interaction of flavin-luciferase complex with oxygen (*Intermediate II* formation) is not influenced by diffusion, probably, due to relatively low rate of flavin binding and high diffusion rate of the oxygen in comparison with flavin. In increasing viscosity of the medium, both substrate-binding steps are slowed down in a similar way and demonstrated the enhanced effect implying contribution of additional factors, not only diffusion control. Taking into account that viscosity effect on such competitive reaction steps as flavin autooxidation and peroxyflavin intermediate dark decay did not exhibit any enhancement, as it was shown earlier in [28], we can conclude that higher viscosity provides no kinetic advantages for the “light” reaction pathways.

The major difference between the two cosolvents was found in the influence on the rate constant of excited state intermediate formation (*Intermediate III*): the presence of sucrose increases catalytic constant up to 5-fold, while it remains the same in glycerol. To shed light on the mechanisms of the cosolvents influence on bioluminescence reaction, we performed molecular dynamics simulations of bacterial luciferase in explicit solvent. It allowed detecting, firstly, that, unlike the sucrose ones, the glycerol molecules penetrate into the active site cavity. Secondly, it appeared that high concentration of sucrose facilitates a conformational change of functionally important αGlu175 of bacterial luciferase. The observed preferable orientation of the αGlu175 side chain could be responsible for a proper conformation of the active site that leads to a higher catalytic activity in sucrose solutions.

Additionally, to check if the difference of glycerol and sucrose action is caused by the size of the cosolvent or by its chemical nature, we studied the bioluminescence reaction kinetics in solution with sorbitol and glucose. Similar effects were obtained for these cosolvents, which are close in size, but are also polyol and carbohydrate like glycerol and sucrose. It indicates that molecular size plays a more important role in the media effects than the chemical nature of the viscogenic agent.

Integrating all the results of our study, we can conclude that the apparent inhibition effect of glycerol on the bacterial luciferase reaction, which is manifested in reduced peak intensity and overall quantum yield, is only a result of slowing down the substrate binding. In other words, glycerol has no effect on the catalytic constant of bacterial luciferase. A different influence on the reaction was found for sucrose: it also decreases substrates binding rates but additionally induces the increase of catalytic efficiency of the enzyme. The possible mechanism of such action is the change in mobility of water molecules in the active site gorge of luciferase caused by location of sucrose molecules out of this area. Only the detailed analysis of individual reaction steps allowed understanding the origins of the observed viscosity effects.

Thus, accessibility or inaccessibility of the enzyme active site for cosolvent molecules, generally determined by their hydrodynamic radii, can significantly change the overall impact of viscous media on the efficiency of enzymatic reaction. We suppose this factor is necessary to consider when interpreting the effects of various model and cytomimetic media on biochemical processes.

## Figures and Tables

**Table 1:** The calculated rate constants for the separate stages of the reaction, catalyzed by bacterial luciferase, in viscous media.

Rate Constant *	Buffer	Cosolvent	10%	20%	30%	40%	$\delta {\;}^{\&}$
$k_{d}$${}^{\#}$, s${}^{-1}$	7.85	Glycerol	7.50	5.05	3.66	2.60	0.81 ± 0.05
		Sucrose	5.69	4.25	3.29	2.64	
$k_{\mathrm{dd}}$${}^{\#}$, s${}^{-1}$	0.15	Glycerol	0.15	0.15	0.12	0.10	0.84 ± 0.11
		Sucrose	0.15	0.12	0.09	0.05	0.82 ± 0.06
$k_{1}$, M${}^{-1}$s${}^{-1}$	1.55	Glycerol	1.06	0.62	0.38	0.19	1.62 ± 0.04
		Sucrose	1.21	0.82	0.47	0.29	0.94 ± 0.03
$k_{2}$, s${}^{-1}$	400	Glycerol	398	403	403	395	N/A
		Sucrose	401	403	393	402	N/A
$k_{3}$, M${}^{-1}$s${}^{-1}$	0.42	Glycerol	0.53	0.30	0.21	0.10	1.21 ± 0.35
		Sucrose	0.31	0.18	0.06	0.02	1.85 ± 0.15
$k_{-3}$, s${}^{-1}$	10.90	Glycerol	8.95	7.50	5.42	5.30	0.59 ± 0.07
		Sucrose	7.02	9.07	9.40	4.67	N/A
$k_{4}$, s${}^{-1}$	0.46	Glycerol	0.45	0.47	0.43	0.42	N/A
		Sucrose	0.38	0.64	1.69	5.13	N/A

* The constants are designated in accordance with the Figure 1. ${}^{\#}$ The values of $k_{d}$ and $k_{\mathrm{dd}}$ have been taken from [28] and fixed during the fitting session. The values for 30% glycerol have been obtained by interpolation. ${}^{\&}$ is an exponent in the power law dependence. N/A means the absence of the power law dependence.

## Data Availability

The data presented in this study are available on request from the corresponding author.

## Supplementary Materials

The following are available online at https://www.mdpi.com/article/10.3390/ijms22168827/s1.
