# Supplementary material for: Mechanisms of Viscous Media Effects on Elementary Steps of Bacterial Bioluminescent Reaction
Source: Int J Mol Sci. 2021 Aug 17;22(16):8827. doi: 10.3390/ijms22168827 (PMC8396235; doi:10.3390/ijms22168827)
Supplement: Supplementary file 1 [file ijms-22-08827-s001.zip › SupplementaryMaterials.pdf]

Mechanisms of viscous media effects on elementary steps

of bacterial bioluminescent reaction

Lisitsa A. E.[1], Sukovatyi L. A.[1], Bartsev S.I.[1,2], Deeva A.A.[1], Kratasyuk V.A.[1,2] and Nemtseva E.V.[1,2]

[1]Siberian Federal University, 660041, Svobodny 79, Krasnoyarsk, Russia

[2]Institute of Biophysics SB RAS, 660036, Akademgorodok 50/50, Krasnoyarsk, Russia

Supplementary Material

Content

Figure S1: The stages of the reaction, catalyzed by bacterial luciferase and details of mathematical modelling...3

Figure S2: An example of kinetic curves of bioluminescent reaction catalyzed by bacterial luciferase in buffer..5

Details of molecular dynamics simulation 6

Table S1: The number of water and cosolvent molecules in the systems used for MD simulations.......................6

Figure S3: Kinetic curves of bioluminescent reaction with 10, 20, 30 and 50 μM of decanal in media.................7

Figure S4: Dependence of kinetic characteristics on media viscosity for reaction with 10 (A), 20 (B), 30 (C) and 50 (D) μM of decanal...9

Figure S5: The fragments of sequence alignment of *V. harveyi* and *P. leiognathi* luciferase with the residues forming active site cavity 11

Figure S6: Relative frequency (RF) of side chain conformations of selected residuals during molecular dynamics simulation at various concentrations of cosolvents 12

Figure S7: Solvent accessible surface area (SASA) of functionally important amino acids of the bacterial luciferase α-subunit in glycerol (A) and sucrose (B) solutions..14

Figure S8: The hydrogen bond occupancy for indicated atoms of the residues involved in flavin binding..........15

Figure S9: The hydrogen bond occupancy for side chains of the residues involved in aldehyde binding............16

References 17

A

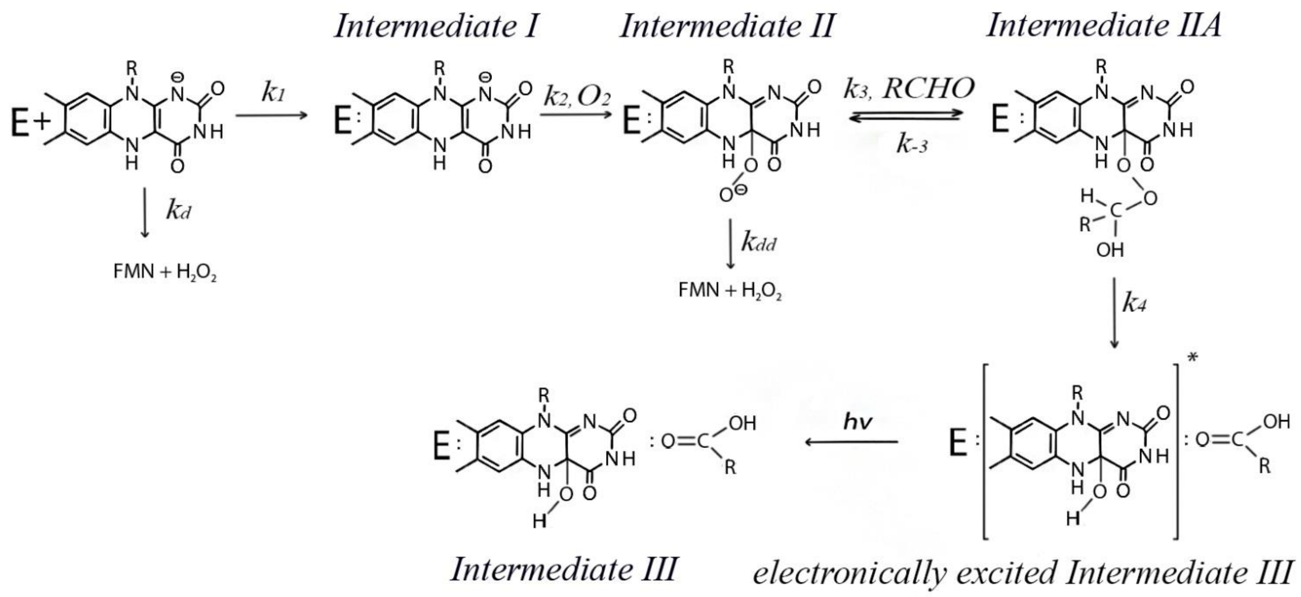

$$
\begin{cases}
\frac{dF}{dt} = -k_1 FL - k_d F \\
\frac{dL}{dt} = -k_1 FL \\
\frac{dI_1}{dt} = k_1 FL - k_2 I_1 \\
\frac{dI_2}{dt} = k_2 I_1 - k_{dd} I_2 - k_3 I_2 A + k_{-3} I_{2A} \\
\frac{dI_{2A}}{dt} = k_3 I_2 A - k_{-3} I_{2A} - k_4 I_{2A} \\
\frac{dI_3}{dt} = k_4 I_{2A} \\
\frac{dA}{dt} = k_{-3} I_3 - k_3 I_2 A
\end{cases}
$$

B

C

$$\Delta_I = \sum_5 \sum_{i=1}^{N} \left(I_{model} - I_{experiment}\right)^2$$

D

$$\delta_I = \frac{\Delta_I}{N} : 5$$

The figure continues on the next page

Figure S1: The stages of the reaction, catalyzed by bacterial luciferase where E – is enzyme, (*) - electronically excited intermediate (A). The set of the ordinary differential equations taken for the modeling of bioluminescence kinetics of reaction catalyzed by bacterial luciferase (B). The formula of the absolute (C) and relative (D) errors minimized by the program during the fitting of kinetic curves (least-squares method); the formula of the relative error of the modelled curves in arbitrary units (E) and percentage (F).

E

$$\Delta_I = \sum_5 \sum_{i=1}^{N} \left| \frac{I_{model} - I_{experiment}}{I_{experiment}} \right|$$

F

$$\delta_I = \left(\frac{\Delta_I}{N} : 5 \right) \cdot 100\%$$

Figure S1: (Continuation) The stages of the reaction, catalyzed by bacterial luciferase where E – is enzyme, (*) - electronically excited intermediate (A). The set of the ordinary differential equations taken for the modeling of bioluminescence kinetics of reaction catalyzed by bacterial luciferase (B). The formula of the absolute (C) and relative (D) errors minimized by the program during the fitting of kinetic curves (least-squares method); the formula of the relative error of the modelled curves in arbitrary units (E) and percentage (F).

The list of designations:

- *F* – reduced flavin mononucleotide
- *L* – bacterial luciferase
- I_1 – *Intermediate I* (complex of luciferase with reduced flavin mononucleotide)
- I_2 – *Intermediate II* (complex of luciferase with C(4a)-hydroperoxyflavin)
- I_{2A} – *Intermediate IIA* (complex of luciferase with C(4a)-peroxyflavin hemiacetal)
- I_3 – *Intermediate III* (complex of luciferase with electronically excited C(4a)-hydroxyflavin)
- *A* – aldehyde
- I_{model} and $I_{experiment}$ – the intensity at each time step in the modeled and experimental kinetic curve, respectively

A

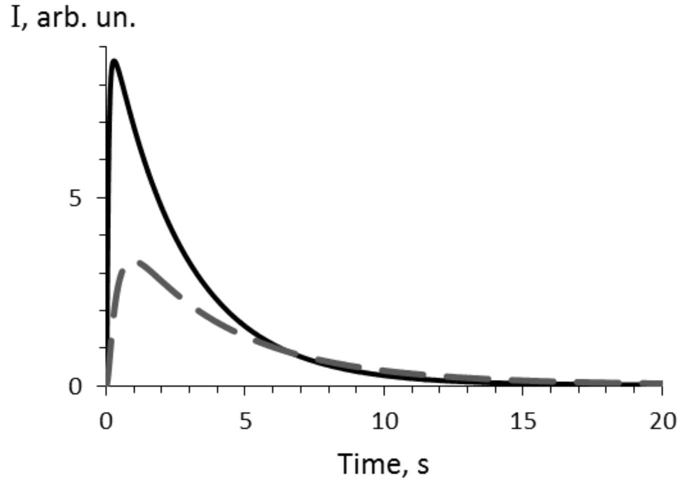

B

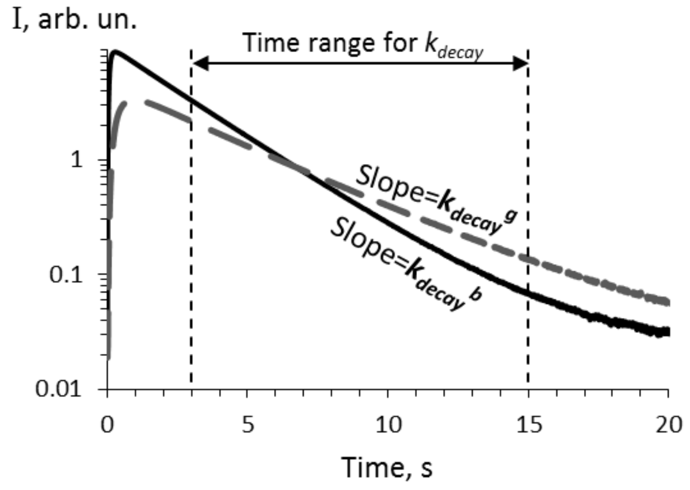

C

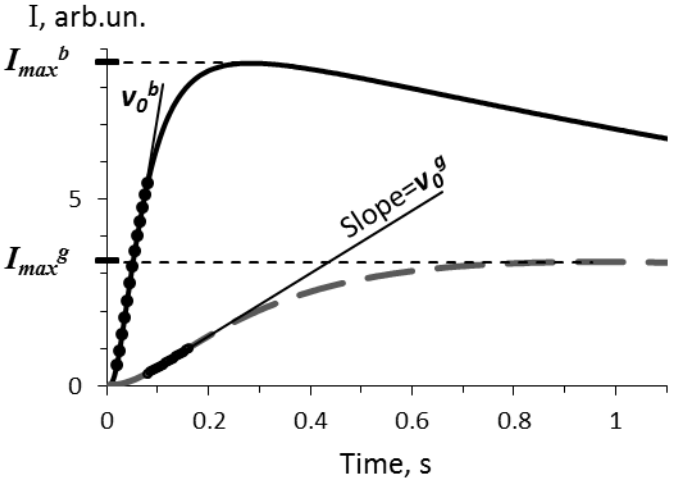

Empirical kinetic parameters

1) The area under the kinetic curve gives the total quantum yield (Q*):

$$Q^* = \sum_{t_i=0}^{20} I(t_i)$$

2) The indicator of exponential function, approximating the kinetic curve in the range of 3-15 s, gives the decay constant (k_{decay}):

$$I(t) = Const1 \cdot e^{-k_{decay}t} + Const2$$

The relation of k_{decay} with the rate constants of reaction stages [1]:

$$k_{decay} = \frac{k_{dd} + k_4 \cdot (\frac{k_3}{k_{-3}}) \cdot A}{1 + (\frac{k_3}{k_{-3}}) \cdot A}$$

3) Maximum bioluminescent signal gives peak intensity I_{max}

4) The slope of the starting linear part of the kinetic curve gives an initial velocity v_0:

$$I(t) = v_0 \cdot t + Const$$

Figure S2: An example of kinetic curves of bioluminescent reaction catalyzed by bacterial luciferase in buffer (black solid line) and in 40% glycerol solution (gray dashed line): full curve in linear scale for I (A), full curve in semilogarithmic scale for I (B), and the rising phase of the curves during 1.1 s (C). Decanal concentration was 50 μM. Superscripts *b* and *g* refer to buffer and glycerol correspondently. Markers (●) refer to the linear part of the kinetic curve. The methods of the empirical kinetic parameters calculation are described in the box on the right.

Details of molecular dynamics simulation

Table S1: The number of water and cosolvent molecules in the systems used for MD simulations.

Systems			Number of water molecules	Number of cosolvent molecules
№	Cosolvent concentration, %	Cosolvent type		
1	0	Glycerol	40821	0
		Sucrose		
2	10	Glycerol	36445	799
3		Sucrose	37443	215
4	20	Glycerol	32289	1597
5		Sucrose	34136	430
6	30	Glycerol	28208	2396
7		Sucrose	30819	645
8	40	Glycerol	24323	3194
9		Sucrose	27589	859

Details of molecular dynamics

Molecular dynamics simulation was performed using GROMACS 5.1.4 package [2]. The crystal structure of *Vibrio harveyi* bacterial luciferase (PDB ID: 3FGC) [3] was initial for all the simulations. All ions, crystal water, and FMN were removed before solvation. The missing segment of the α-subunit mobile loop (corresponding to residues 283–290) was reconstructed by MODELLER in UCSF CHIMERA software package [4,5]. The protein was solvated in a cubic box with explicit solvent extending at least 12 Å from the protein to the edges of the box. Nine systems were prepared: luciferase molecule surrounded by water molecules and mixtures with glycerol or sucrose molecules adjusted to simulate the media with 10, 20, 30, 40 w/w % of cosolvent [Table S1]. The CHARMM36 parameters were used to model protein atoms and the TIP3P water model [6,7]. The force field parameters for glycerol and sucrose were obtained from CHARMM topology files [8]. Each system was made electrostatically neutral with randomly placed 36 potassium counterions.

The solvated protein system was minimized with steepest descent method (maximum force of 1000.0 kJ/mol). The cutoff distance for the short-range non bonded interactions was 12 Å and electrostatic interactions were treated using the particle mesh Ewald method [9]. An integration step of 2.0 fs was used, and bonds were constrained with the LINCS algorithm [10]. For all equilibration steps and simulations the periodic boundary conditions were used. The minimized system was heated and equilibrated at 300 K for 5 ns in the NVT ensemble with the restrained protein. Following 10 ns NPT equilibration was carried out with protein heavy atom restriction. Due to removing FMN from active site and homology base reconstruction of luciferase's mobile loop additional 10 ns of NPT ensemble unconstrained equilibration was performed. Equilibration was carried out at 300 K temperature using V-rescale thermostat and at 1 bar pressure using the Parrinello-Rahman barostat [11,12].The MD simulation was carried out for 40 ns at a constant temperature of 300 K and 1 atm pressure (NPT ensemble), and coordinates were recorded every 10 ps. To collect statistical data, three independent MD runs of each system were performed.

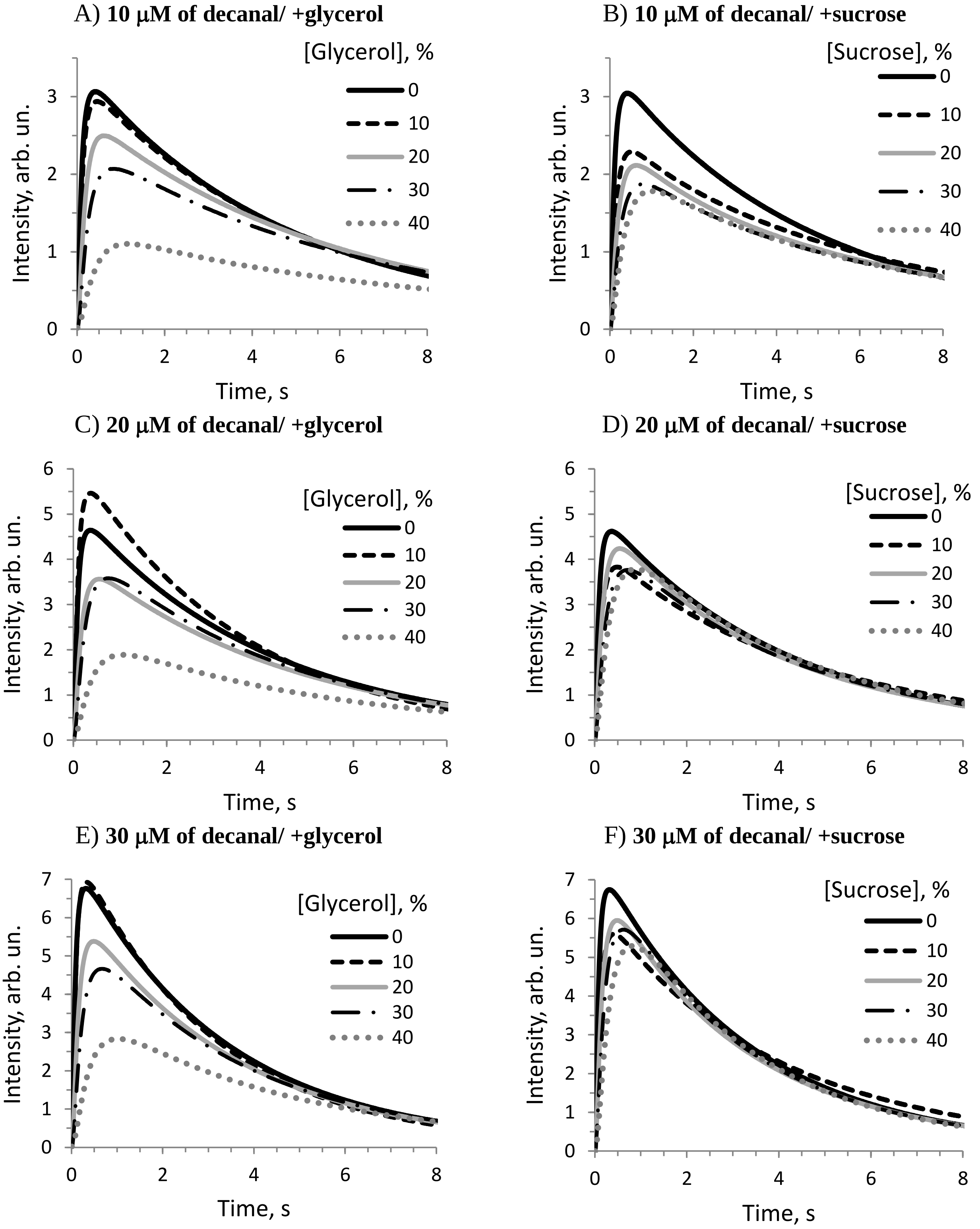

The figure continues on the next page

Figure S3: Kinetic curves of bioluminescent reaction with 10, 20, 30 and 50 μM of decanal in media with glycerol (A, C, E, G) and sucrose (B, D, F, H).

G) **50 μM of decanal/ +glycerol**

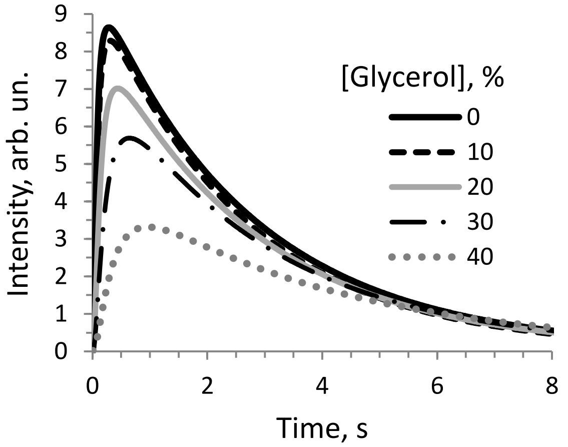

H) **50 μM of decanal/ +sucrose**

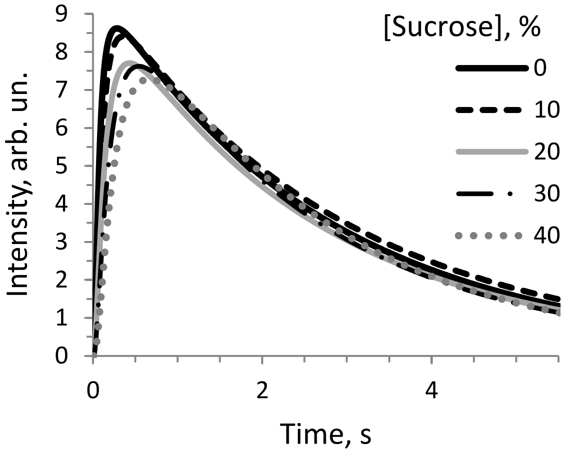

Figure S3: (Continuation) Kinetic curves of bioluminescent reaction with 10, 20, 30 and 50 μM of decanal in media with glycerol (A, C, E, G) and sucrose (B, D, F, H).

A) **10 μM of decanal**

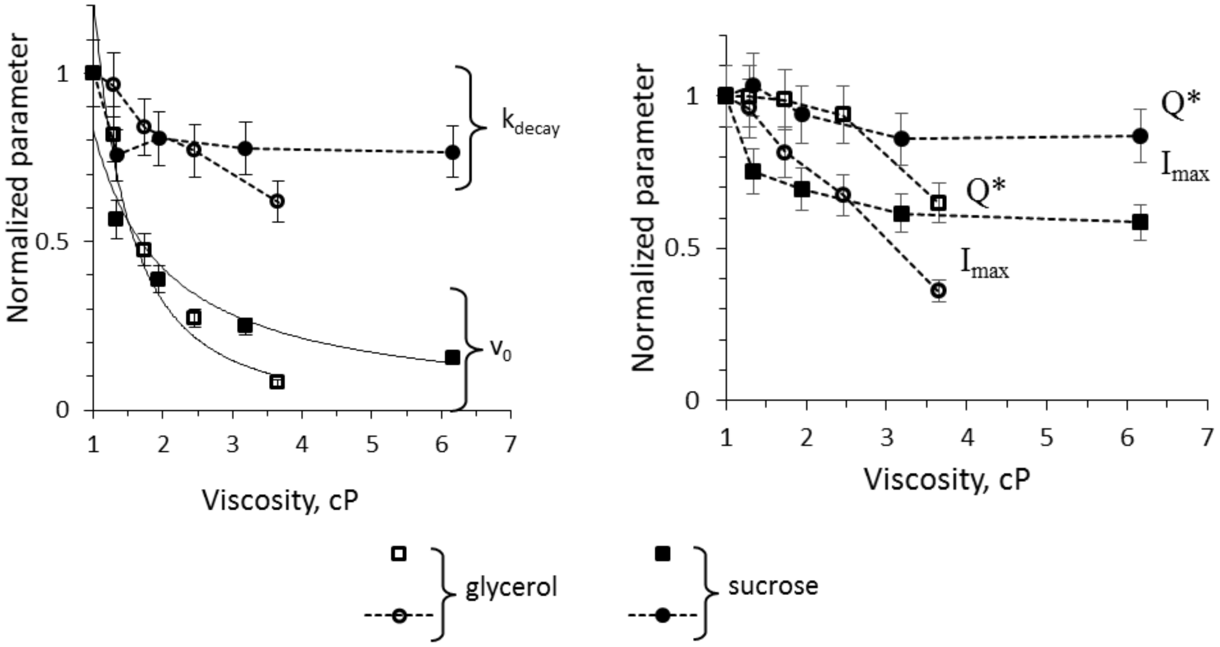

B) **20 μM of decanal**

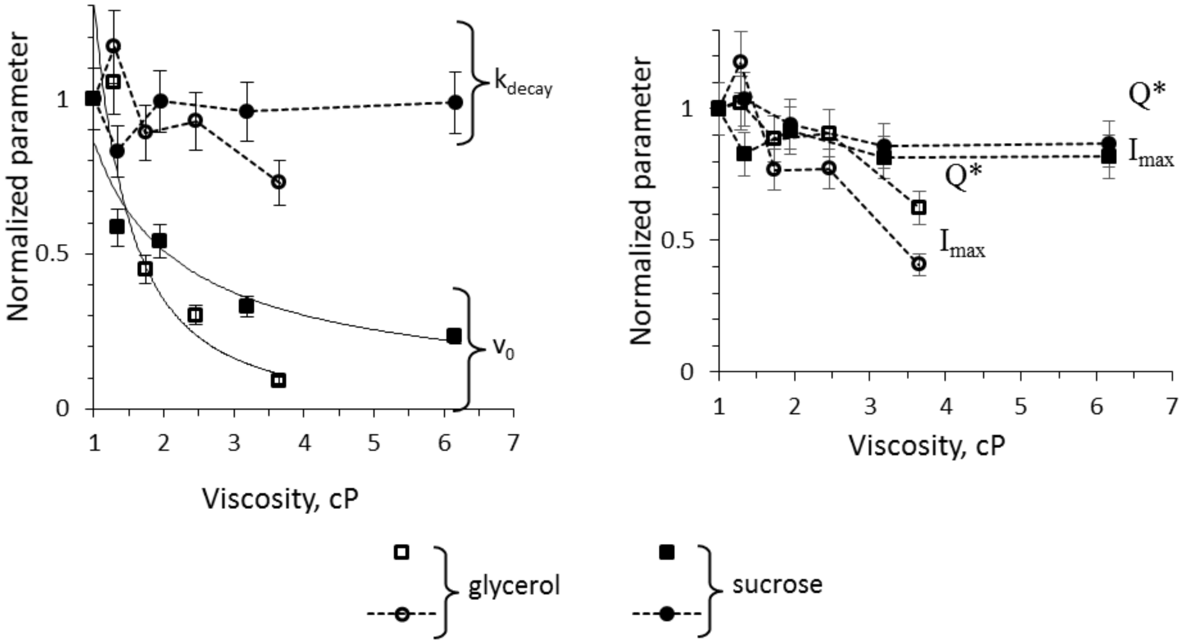

The figure continues on the next page

Figure S4: Dependence of kinetic characteristics on media viscosity for reaction with 10 (A), 20 (B), 30 (C) and 50 (D) μM of decanal

C) **30 μM of decanal**

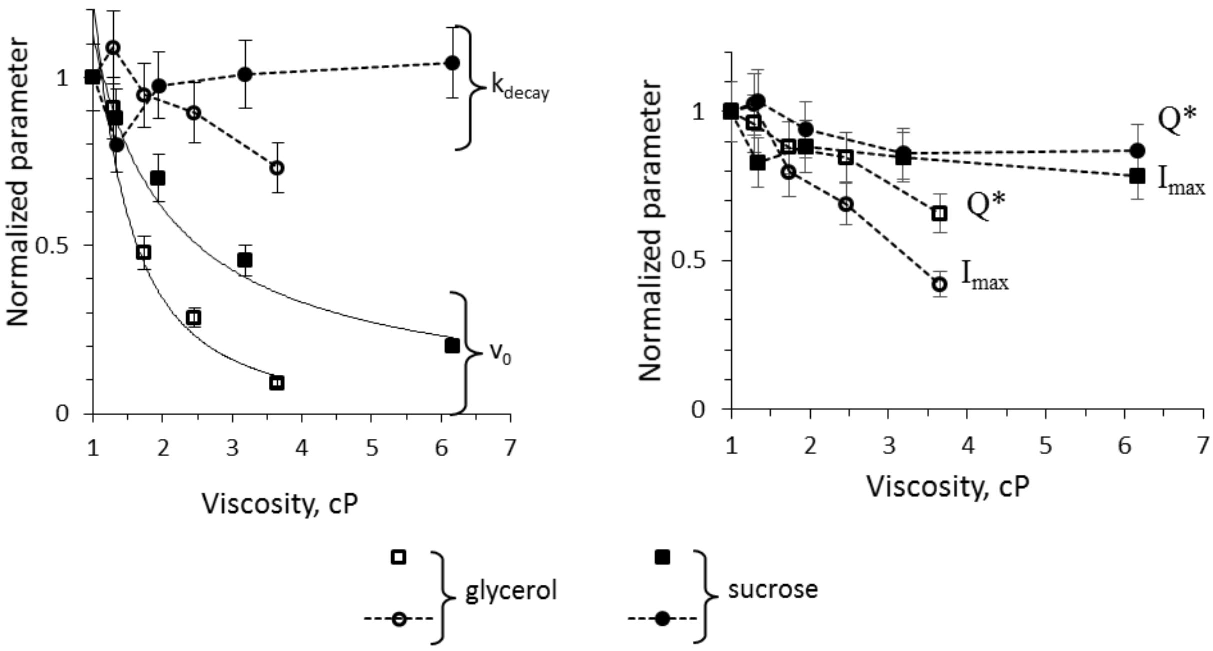

D) **50 μM of decanal**

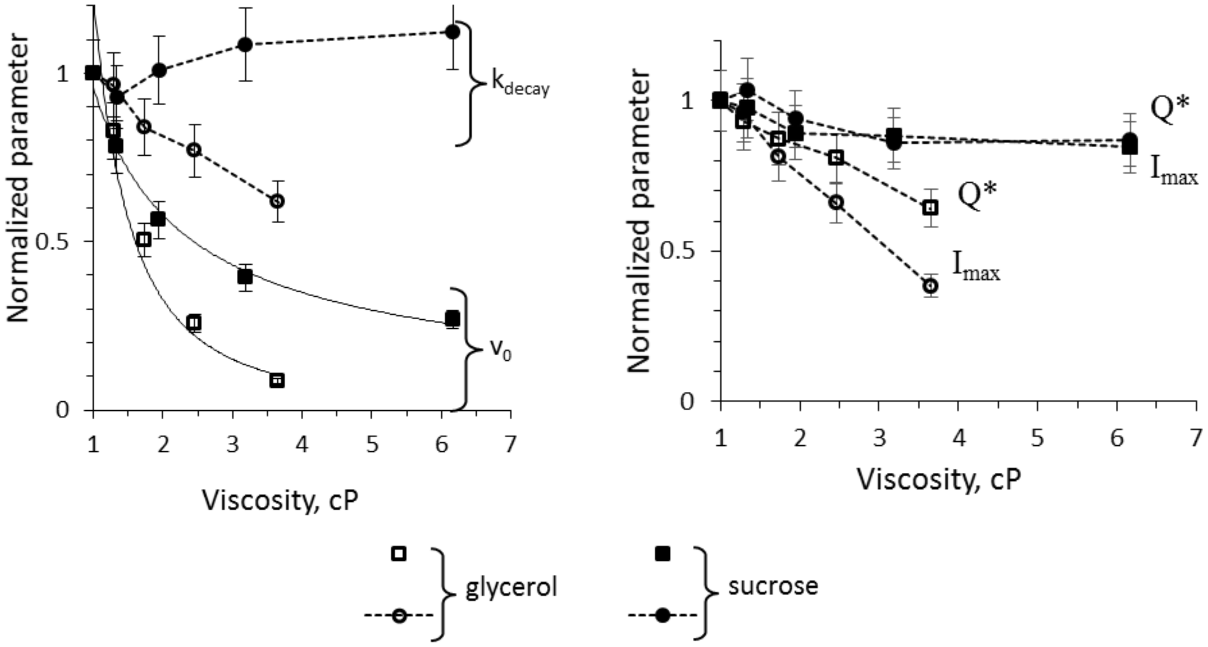

Figure S4: (Continuation) Dependence of kinetic characteristics on media viscosity for reaction with 10 (A), 20 (B), 30 (C) and 50 (D) μM of decanal.

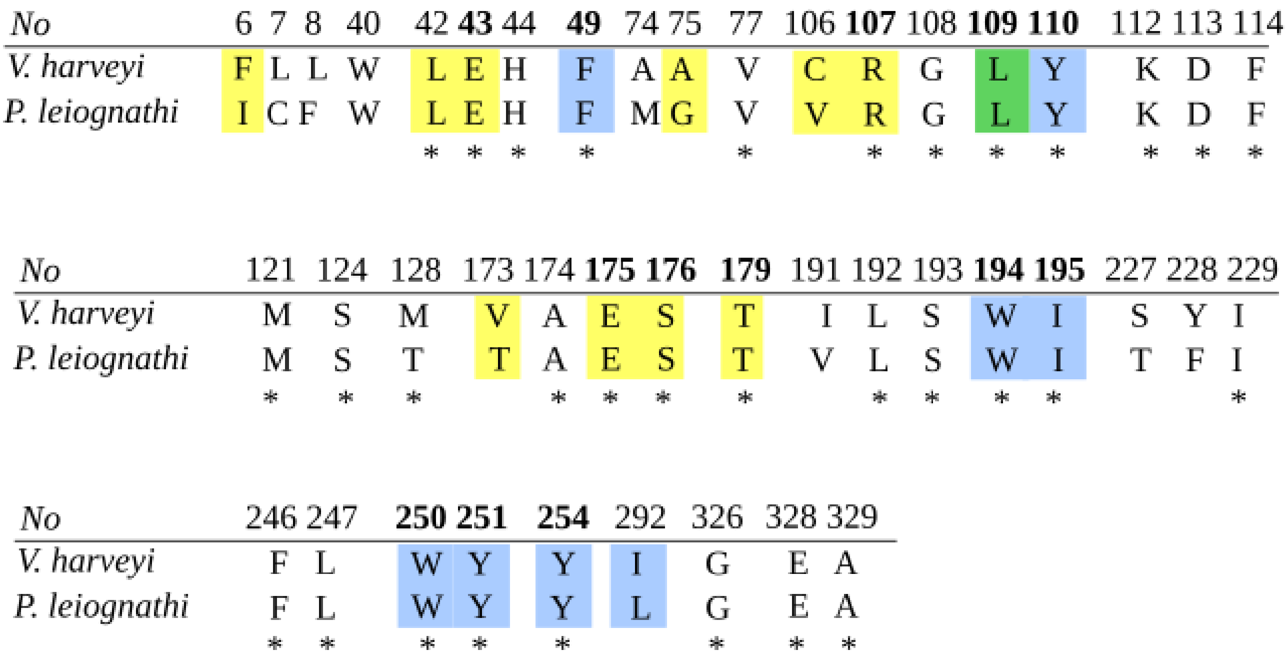

Figure S5: The fragments of sequence alignment of *V. harveyi* and *P. leiognathi* luciferase with the residues forming active site cavity (as determined by CASTp web-service). Flavin and aldehyde binding sites are colored yellow and blue, respectively (according to [3] and [13]). Leu109 is marked by green because it participates in binding of both substrates. The residues identical among 21 sequences of bacterial luciferases from different species are marked by asterisk [14]. The numbers of the residuals studied in the current work are in bold.

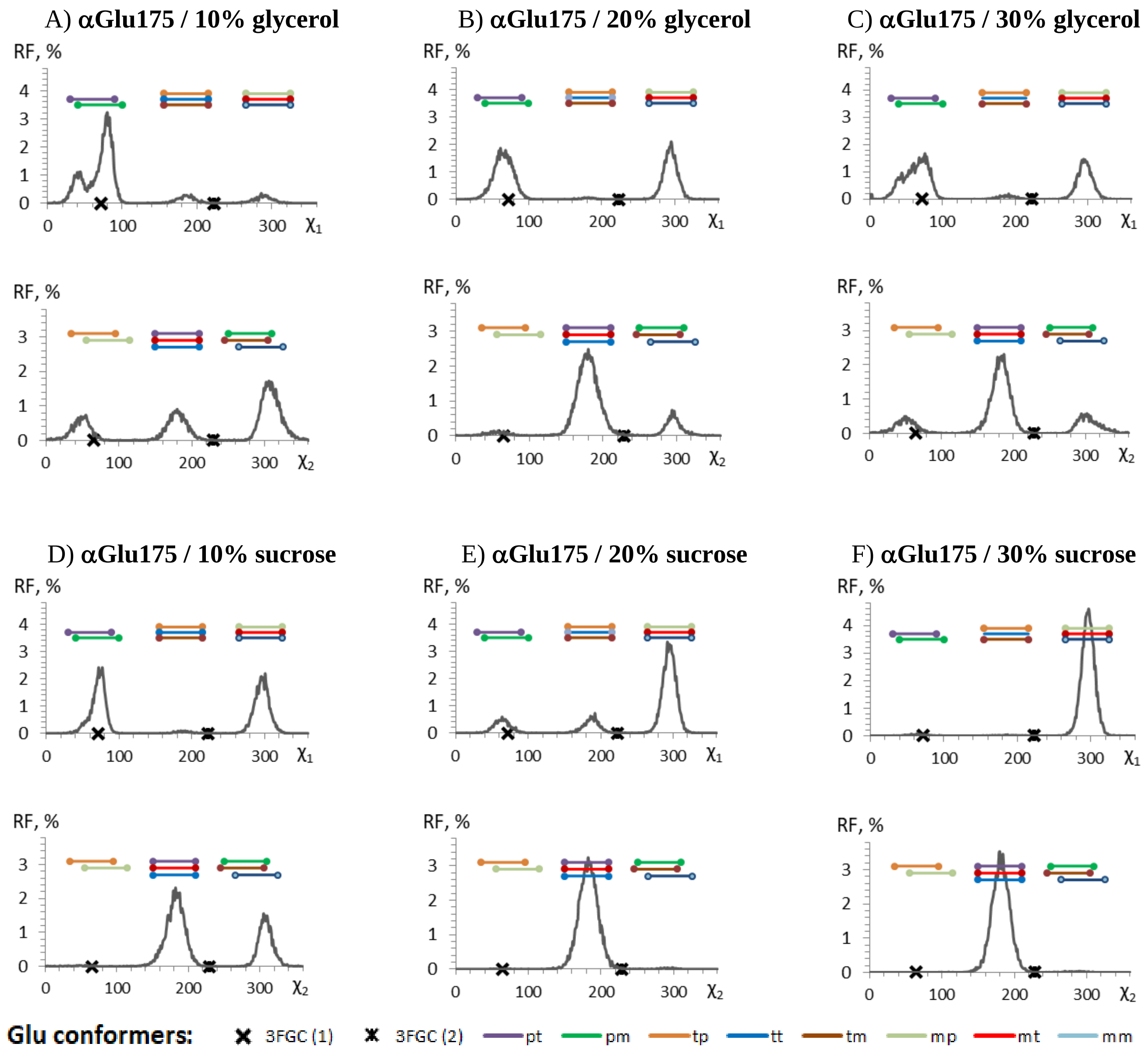

The figure continues on the next page

Figure S6: Relative frequency (RF) of side chain conformations of selected residuals during molecular dynamics simulation at various concentrations of cosolvents: A-G – χ_1 (upper panel) and χ_2 (lower panel) dihedrals of αGlu175; H-M – χ_1 dihedral of αSer176. By the colored straight lines the dihedral intervals of known conformations are shown (according to [15]). By the crosses the conformers found in luciferase crystal (PDB ID: 3FGC) are indicated [3].

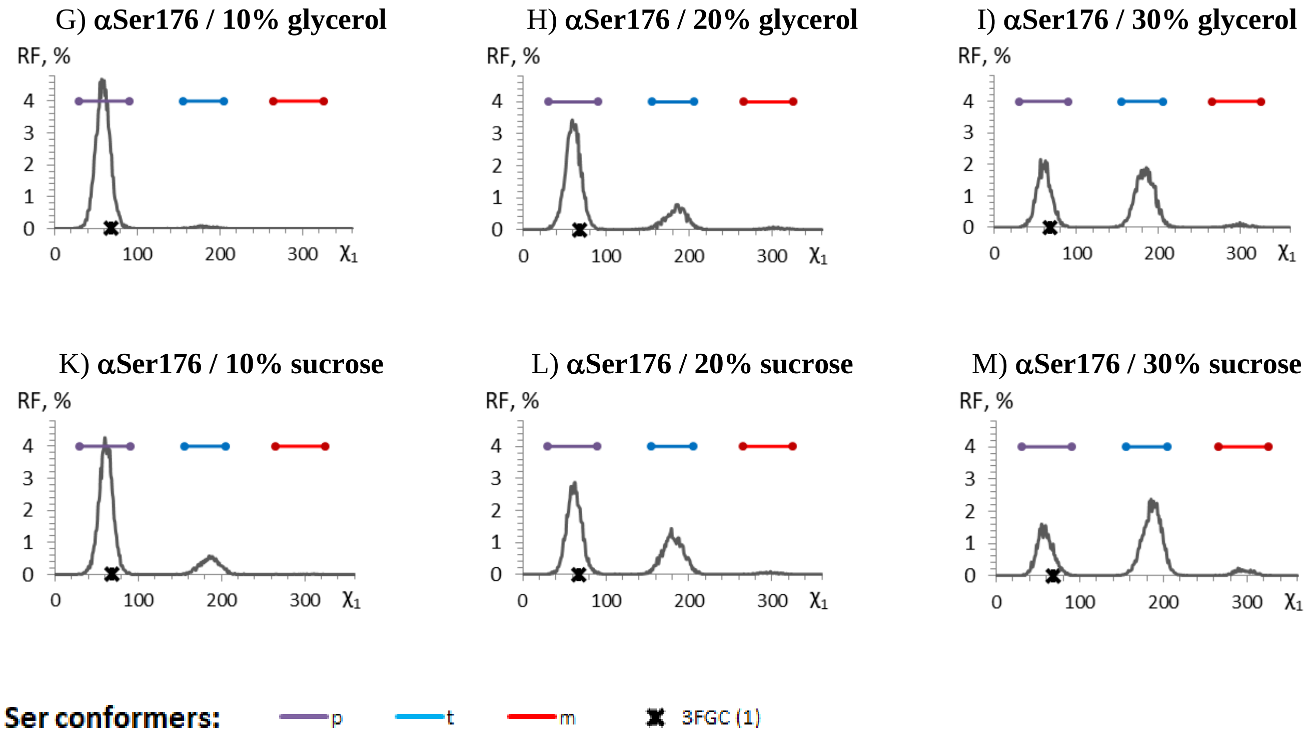

Figure S6: (Continuation) Relative frequency (RF) of side chain conformations of selected residuals during molecular dynamic simulation at various concentrations of cosolvents: A-G – χ_1 (upper panel) and χ_2 (lower panel) dihedrals of αGlu175; H-M – χ_1 dihedral of αSer176. By the colored straight lines the dihedral intervals of known conformations are shown (according to [15]). By the crosses the conformers found in luciferase crystal (PDB ID: 3FGC) are indicated [3].

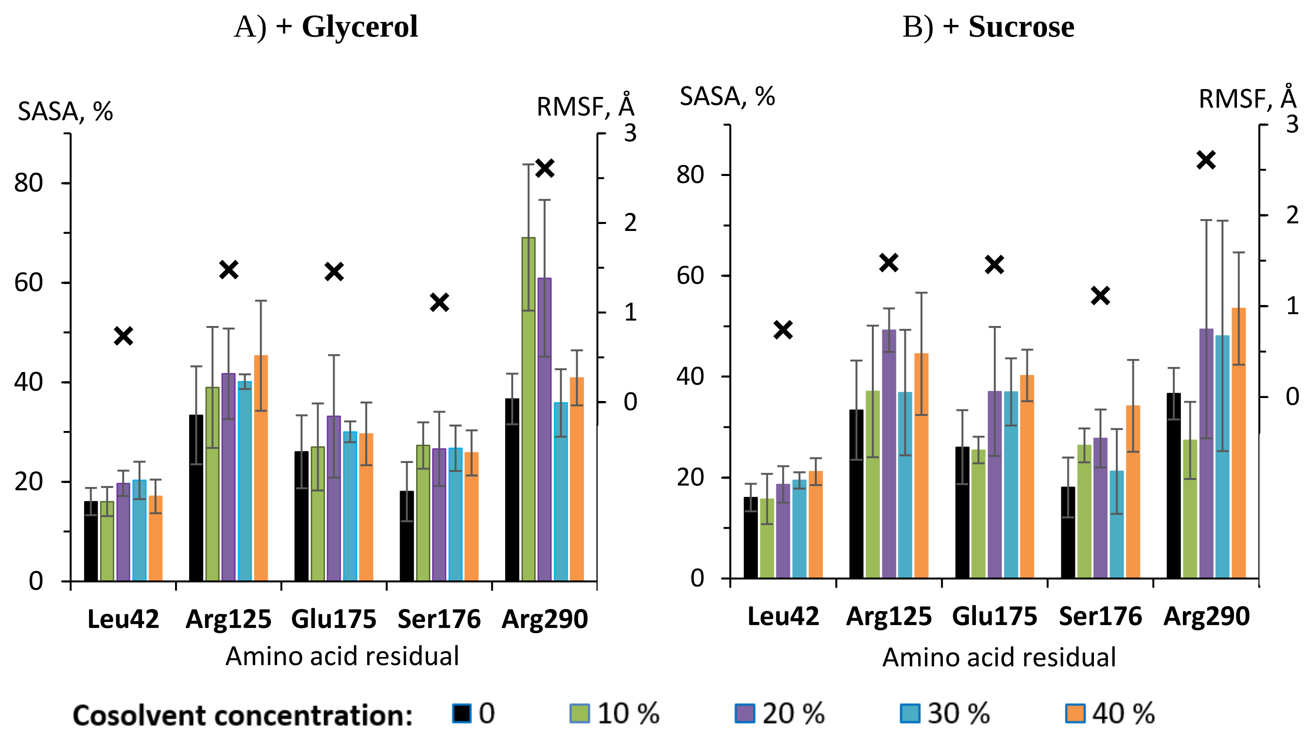

Figure S7: Solvent accessible surface area (SASA) of functionally important amino acids of the bacterial luciferase α-subunit in glycerol (A) and sucrose (B) solutions. SASA is shown as a percentage of maximal possible solvent accessible surface area of each amino acid [16]. With the cross the average RMSF value of amino acid side chain is shown.

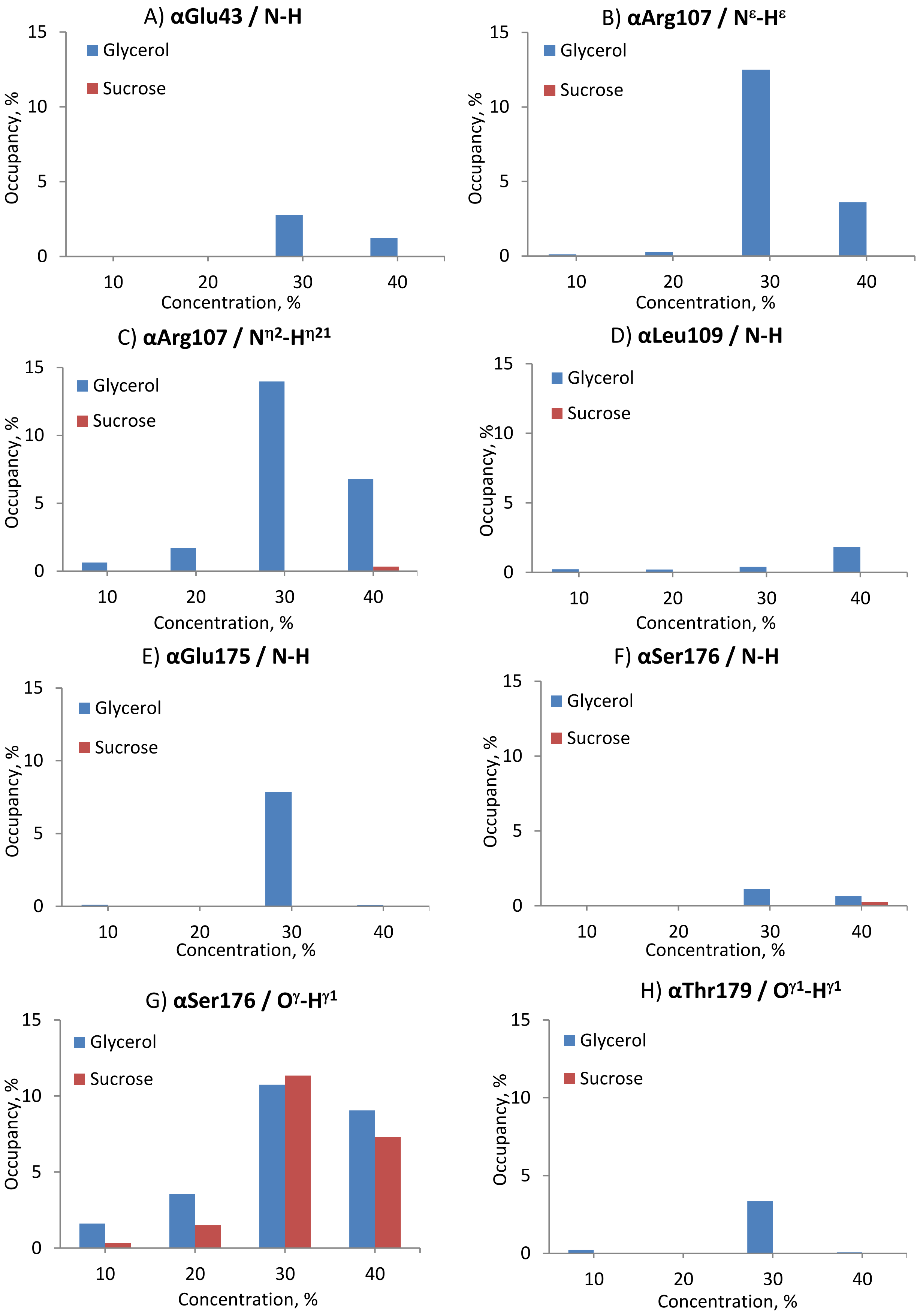

Figure S8: The hydrogen bond occupancy for indicated atoms of the residues involved in flavin binding. The cut off Donor-Acceptor distance was 3.5 Å and Hydrogen-Donor-Acceptor angle 30°. The fractional occupancy was calculated throughout aggregated data of three independent MD-simulations: A) α43Glu/N-H, B) α107Arg/N^{ε}-H^{ε}, C) α107Arg/$N^{\eta 2}$-$H^{\eta 21}$, D) α109Leu/N-H, E) α175Glu/N-H, F) α176Ser/N-H, G) α176Ser/O^{γ}-$H^{\gamma 1}$, H) αThr179/ $O^{\gamma 1}$-$H^{\gamma 1}$.

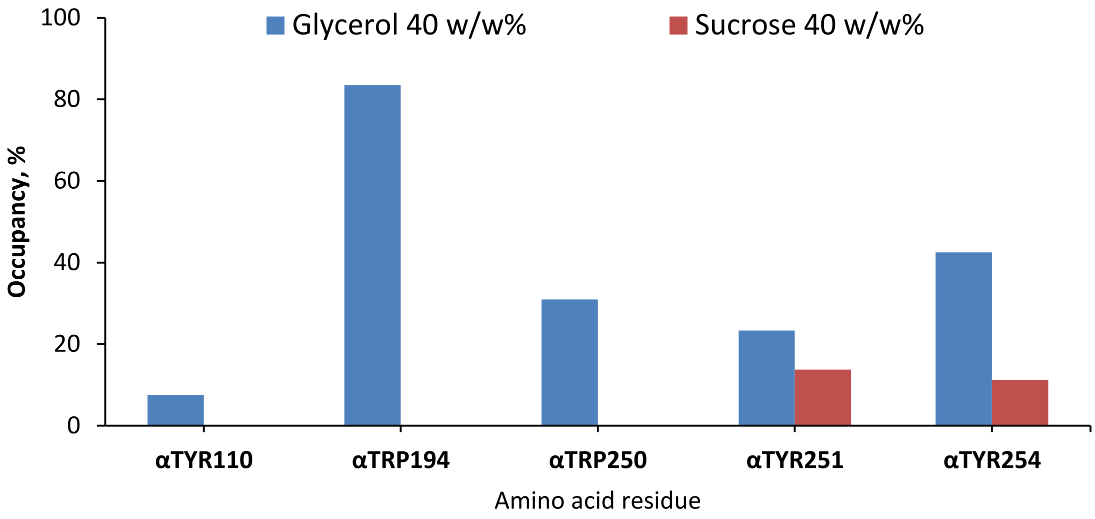

Figure S9: The hydrogen bond occupancy for side chains of the residues involved in aldehyde binding [13]. The cut off Donor-Acceptor distance was 3.5 Å and Hydrogen-Donor-Acceptor angle 30°. The fractional occupancy was calculated throughout aggregated data of three independent MD-simulations. The atoms capable of forming hydrogen bonds (both as donor and as acceptor): αTyr110/O^{η}-H^{η}, αTrp194/$N^{\varepsilon 1}$-$H^{\varepsilon 1}$, αTrp250/$N^{\varepsilon 1}$-$H^{\varepsilon 1}$, αTyr251/O^{η}-H^{η}, αTyr254/O^{η}-H^{η}.

References

1. Cline, T. W., & Hastings, J. W. (1972). Mutationally altered bacterial luciferase. Implications for subunit functions. *Biochemistry, 11*(18), 3359-3370.
2. Van Der Spoel, D., Lindahl, E., Hess, B., Groenhof, G., Mark, A. E., &Berendsen, H. J. (2005). GROMACS: fast, flexible, and free. Journal of computational chemistry, 26(16), 1701-1718.
3. Campbell, Z. T., Weichsel, A., Montfort, W. R., & Baldwin, T. O. (2009). Crystal structure of the bacterial luciferase/flavin complex provides insight into the function of the β subunit. Biochemistry, 48(26), 6085-6094.
4. Šali, A., & Blundell, T. L. (1993). Comparative protein modelling by satisfaction of spatial restraints. Journal of molecular biology, 234(3), 779-815.
5. Pettersen, E. F., Goddard, T. D., Huang, C. C., Couch, G. S., Greenblatt, D. M., Meng, E. C., &Ferrin, T. E. (2004). UCSF Chimera—a visualization system for exploratory research and analysis. Journal of computational chemistry, 25(13), 1605-1612.
6. Best, R. B., Zhu, X., Shim, J., Lopes, P. E., Mittal, J., Feig, M., &MacKerell Jr, A. D. (2012). Optimization of the additive CHARMM all-atom protein force field targeting improved sampling of the backbone ϕ, ψ and side-chain $\chi 1$ and $\chi 2$ dihedral angles. Journal of chemical theory and computation, 8(9), 3257-3273.
7. Jorgensen, W. L., Chandrasekhar, J., Madura, J. D., Impey, R. W., & Klein, M. L. (1983). Comparison of simple potential functions for simulating liquid water. The Journal of chemical physics, 79(2), 926-935.
8. Guvench, O., Mallajosyula, S. S., Raman, E. P., Hatcher, E., Vanommeslaeghe, K., Foster,T. J., ... & MacKerell Jr, A. D. (2011). CHARMM additive all-atom force field forcarbohydrate derivatives and its utility in polysaccharide and carbohydrate–proteinmodeling. Journal of chemical theory and computation, 7(10), 3162-3180.
9. Darden, T., York, D., & Pedersen, L. (1993). Particle mesh Ewald: An N· log (N) method for Ewald sums in large systems. The Journal of chemical physics, 98(12), 10089-10092.
10. Hess, B., Bekker, H., Berendsen, H. J., &Fraaije, J. G. (1997). LINCS: a linear constraint solver for molecular simulations. Journal of computational chemistry, 18(12), 1463-1472.
11. Bussi, G., Donadio, D., &Parrinello, M. (2007). Canonical sampling through velocity rescaling. The Journal of chemical physics, 126(1), 014101.
12. Parrinello, M.; Rahman, A. Polymorphic transitions in single crystals: A new molecular dynamics method. J. Appl. Phys. (1981), 52, 7182–7190.
13. Aldehyd Tang, Y. Q., Luo, Y., & Liu, Y. J. (2021). Theoretical Study on Role of Aliphatic Aldehyde in Bacterial Bioluminescence. Journal of Photochemistry and Photobiology A: Chemistry, 113446.
14. Deeva, A. A., Temlyakova, E. A., Sorokin, A. A., Nemtseva, E. V., & Kratasyuk, V. A. (2016). Structural distinctions of fast and slow bacterial luciferases revealed by phylogenetic analysis. Bioinformatics, 32(20), 3053-3057.
15. Lovell, S. C., Word, J. M., Richardson, J. S., & Richardson, D. C. (2000). The penultimate rotamer library. Proteins: Structure, Function, and Bioinformatics, 40(3), 389-408.
16. Tien, M. Z., Meyer, A. G., Sydykova, D. K., Spielman, S. J., & Wilke, C. O. (2013). Maximum allowed solvent accessibilites of residues in proteins. PloS one, 8(11), e80635.
